# *In silico* clinical trials for pediatric orphan diseases

**DOI:** 10.1038/s41598-018-20737-y

**Published:** 2018-02-06

**Authors:** A. Carlier, A. Vasilevich, M. Marechal, J. de Boer, L. Geris

**Affiliations:** 1 Biomechanics Section, KU Leuven, Celestijnenlaan 300C, PB 2419, 3000 Leuven, Belgium and Biomechanics Research Unit, University of Liège, Chemin des Chevreuils 1 – BAT 52/3, 4000 Liège 1, Belgium; 2 Prometheus, Division of Skeletal Tissue Engineering, KU Leuven, O&N 1, Herestraat 49, PB 813, 3000 Leuven, Belgium; 3 MERLN Institute for Technology-Inspired Regenerative Medicine, Maastricht University, Universiteitssingel 40, 6229 ER Maastricht, The Netherlands; 4 Skeletal Biology and Engineering Research Center, KU Leuven, O&N 1, Herestraat 49, PB 813, 3000 Leuven, Belgium

## Abstract

To date poor treatment options are available for patients with congenital pseudarthrosis of the tibia (CPT), a pediatric orphan disease. In this study we have performed an *in silico* clinical trial on 200 virtual subjects, generated from a previously established model of murine bone regeneration, to tackle the challenges associated with the small, pediatric patient population. Each virtual subject was simulated to receive no treatment and bone morphogenetic protein (BMP) treatment. We have shown that the degree of severity of CPT is significantly reduced with BMP treatment, although the effect is highly subject-specific. Using machine learning techniques we were also able to stratify the virtual subject population in adverse responders, non-responders, responders and asymptomatic. In summary, this study shows the potential of *in silico* medicine technologies as well as their implications for other orphan diseases.

## Introduction

Orphan diseases are defined as disorders with low prevalence (frequency of fewer than 6.5–10 patients in 10,000 according to the WHO) and inadequate treatment strategies^1^. Although the general awareness has grown over the last three decades^1^, rare-disease research is faced with a number of specific challenges, such as the small number of trained disease experts, difficulties in obtaining research funding due to the limited economic impact, the geographic dispersion of patients which hampers patient recruitment for clinical trials and the need for specialized study designs to deal with the small patient cohorts, and high patient variability in the course of the disease^2^. Moreover, 50% of the patients affected by rare diseases are children^3^, which further complicates clinical research due to the uniqueness of children and particular ethical concerns^4^.

In order to tackle the challenges associated with rare diseases, specific incentives have been put into place to develop therapies, including amongst others tax credits, orphan product development grants and market exclusivity (7 years in the USA, 10 years in the European Union and Japan, and 5 years in Australia)^1,5^. Unfortunately, the high failure rate of drug development, with less than 10% of new compounds that enter clinical trials ultimately arriving to the market^6,7^, leads to high prices of orphan disease drugs and a significant socioeconomic burden^8^. As such, the academic research community and the pharmaceutical industry are developing novel statistical methods for the design and analysis of small-population clinical trials^9^. Moreover, the use of *in silico* models and virtual clinical trials is increasingly being explored to understand the mechanisms underlying orphan diseases and to develop novel therapies^10^.

**Figure 1:**
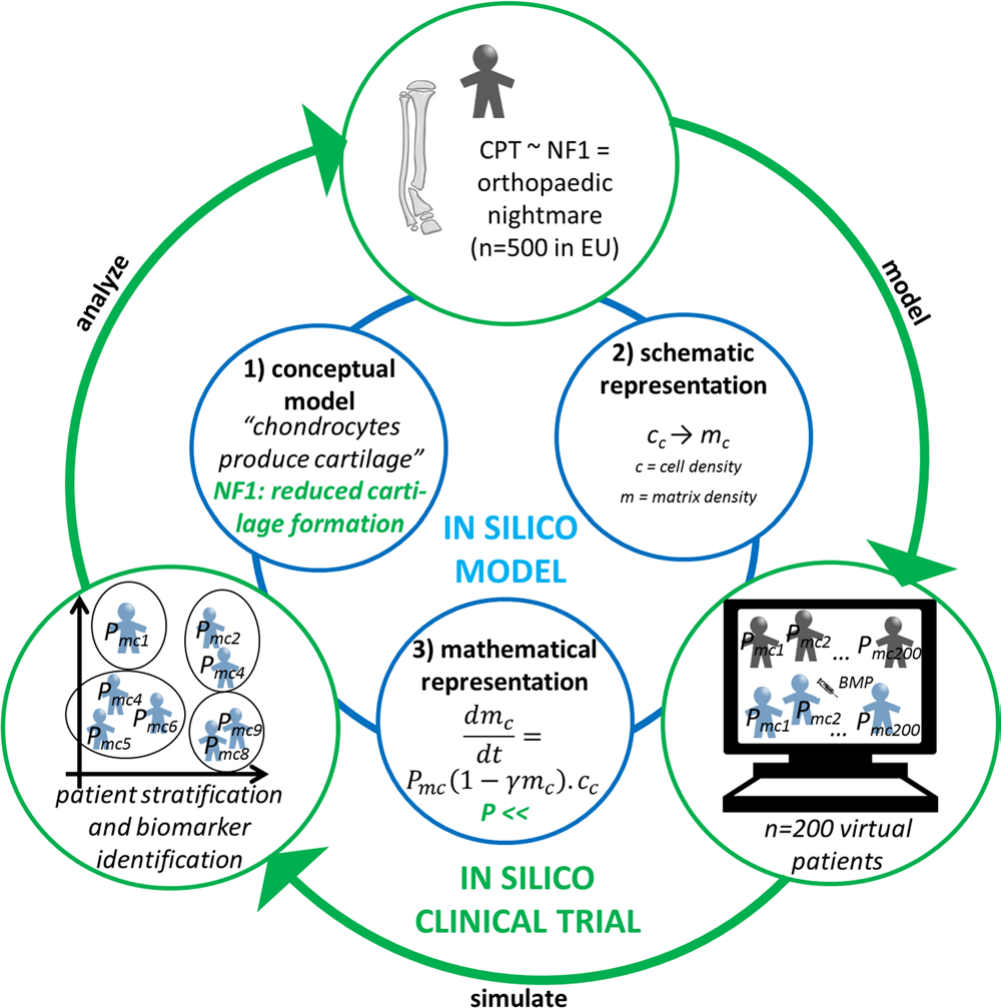
Schematic overview of the *in silico* clinical trial presented in this study. A previously developed mechanistic model of murine bone regeneration was adapted to describe the aberrant cellular behavior of cells affected by NF1 mutation by altering the corresponding parameter values (inner, blue circle: *in silico* model). As an example, the inner circle shows how a conceptual model (1) of cartilage generation can be translated in a schematic (2) and mathematical representation (3). By altering the parameter values, various physiological and pathological behaviors can be represented (indicated in green). From the altered *in silico* model, a cohort of 200 “virtual subjects,” each representing a different parameter combination, was generated. For each virtual subject the bone-healing process was simulated both with BMP treatment and without BMP treatment (outer, green circle: *in silico* clinical trial).

*In silico* models are abstract representations of complex systems in terms of equations or rules and a description of the region (spatially and/or temporally) on which the set of equations is valid^11^. Figure 1 gives a schematic overview of the modeling cycle, where the key components and biological processes are first formulated in a conceptual model (here: cartilage production), which is then translated into a mathematical form. Note that in this example, cartilage is produced at a rate *P*_*mc*_, which is a numerical value that can be tuned to represent normal or pathological chondrocyte behavior. In this way, computational models provide a tool to qualitatively and quantitatively investigate both the individual and combined effects of various treatments on the rare disease phenotype. Moreover, computational models offer a practical, economical, and ethical alternative to *in vivo* experiments by testing a large set of different conditions (e.g., dosing and timing of administration) and selecting the most promising ones. Also, by tuning the rate *P*_*mc*_ (and other parameters in more extensive models), the *in silico* model can be personalized, each unique parameter set representing a unique, “virtual subject.” Since hundreds of different parameter sets can be created *in silico*, a large cohort of virtual subjects can be established, overcoming the limitation of small patient cohorts in rare diseases. Furthermore, the challenges associated with (pediatric) randomized clinical trials are avoided by using the *in silico* model to generate a unique paired data set of both treated and non-treated virtual subjects.

In this study we aim to combine data-driven and mechanistic modeling approaches to illustrate how *in silico* models can contribute to unraveling the mechanisms underlying orphan diseases, with a particular focus on congenital pseudarthrosis of the tibia (CPT) associated with mutations in *Neurofibromatosis Type 1* (*NF1*). CPT is a rare disease which is characterized by a progressive bowing of the tibia and develops into spontaneous fractures in the distal third of the tibia^12^. Typically the bone regeneration is insufficient, resulting in a pseudarthrosis, which is treated by physical excision of the abnormal bone tissue (called the ‘hamartoma’) and internal or external fixation^12^. In recent clinical practice, rhBMP-2 or rhBMP-7 (recombinant human bone morphogenetic protein) is used to enhance the surgical outcome. However, the effectiveness of BMP therapies is still highly debated^13,14^ and the FDA has issued a warning against the use of BMP in skeletally immature patients^15^. As such, the goal of this study is to assess the effect of BMP treatment in an extensive, investigative *in silico* clinical trial using a previously developed mechanistic model of murine bone regeneration^16^ and advanced machine-learning techniques to mine the large set of virtual subject data.

## Results

### The virtual clinical trial predicted the efficacy of treatment

**Figure 2:**
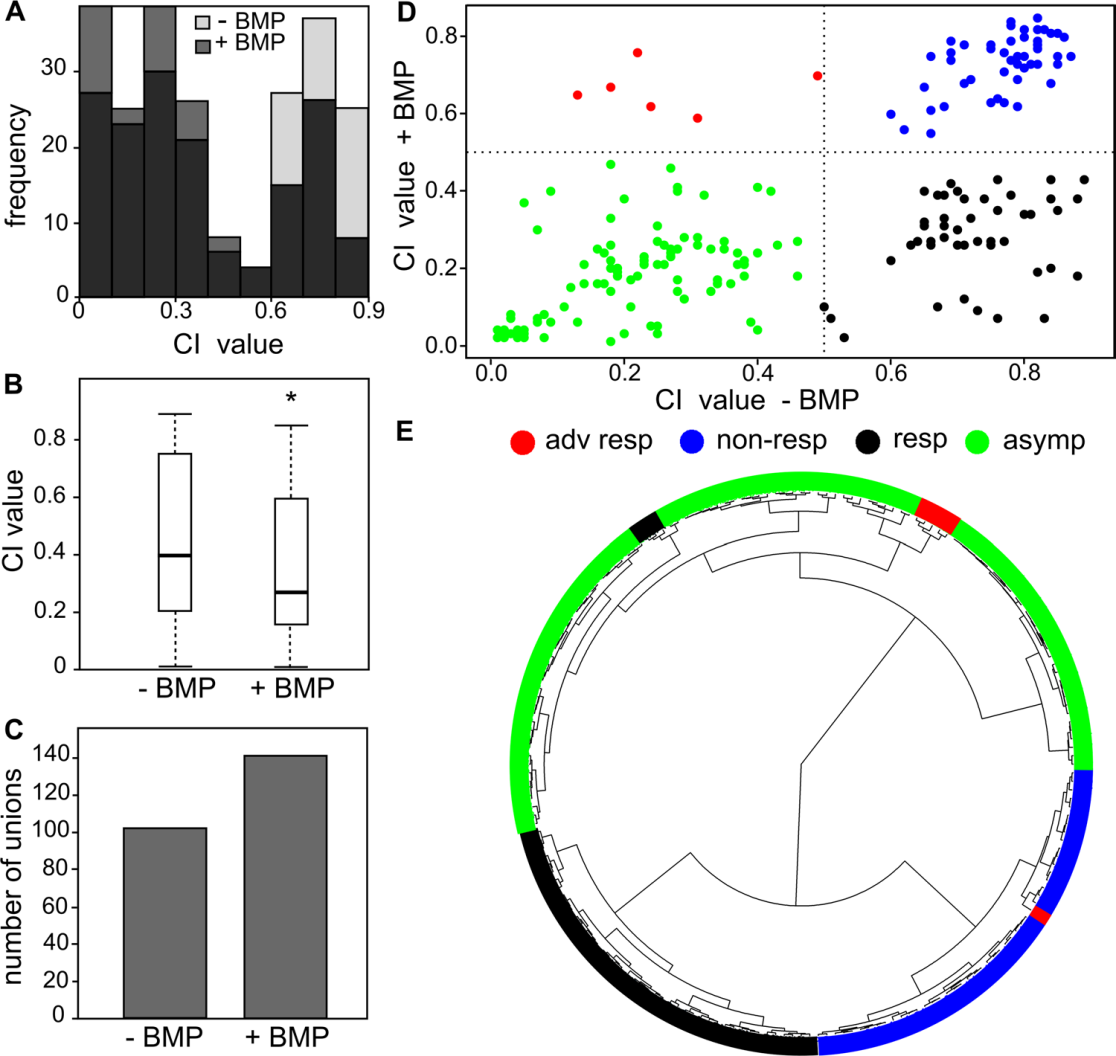
Results of the *in silico* clinical trial of 200 virtual subjects. (**A**) Distribution of the CI values of the treated and non-treated virtual subjects. (**B**) Box plot shows that virtual subjects with BMP treatment had significantly lower CI values (y-axis). *P < 0.01. (**C**) Overview of the amount of bony unions with and without BMP treatment. (**D**) Scatterplot of the treated versus non-treated subjects. Using a cutoff CI of 0.5 (gray dashed lines), the virtual subjects clustered in four classes: adverse responders (red, *n* = 6), non-responders (blue, *n* = 47), asymptomatic (green, *n* = 100), and responders (black, *n* = 47). (**E**) Fanned dendrogram of the treated versus non-treated subjects, classified using Ward hierarchical clustering. Note that the manually defined classes in panel D are also grouped together here.

The degree of severity of CPT was assessed by the complication index (CI), a linear combination of three phenotypic CPT symptoms (see *Methods*). A parameter combination yielding a small CI value reflects a fairly normal fracture-healing process with only minor aspects of CPT (if any). Conversely, a parameter combination resulting in a large CI value represents severely impaired fracture healing reminiscent of the CPT phenotype. The virtual clinical trial predicted a wide range of CI values, corresponding to different degrees of severity by which the pseudarthrosis presents itself, for both the treated and the non-treated group of subjects (Fig. 2A). The group of treated subjects was characterized by a significantly lower mean CI value (Fig. 2B, *p < 0.01) and a higher amount of bony unions (Fig. 2C), indicating a beneficial effect of BMP treatment.

### The virtual clinical trial stratified subjects

To see whether the whole virtual subject population responded similarly to the BMP treatment, we plotted for each subject the results of the treatment versus no treatment (Fig. 2D). Using an arbitrary cut-off of CI value of 0.5, the virtual subjects clustered into four distinct groups: adverse responders (a low CI when untreated and a high CI when treated, red); non-responders (a high CI untreated and with treatment, blue); asymptomatic (a low CI untreated and with treatment, green); and responders (high CI when non-treated and a lower CI when treated, black).

Interestingly, unsupervised machine-learning methods (Ward hierarchical clustering) computationally confirmed the existence of three classes (Fig. 2E): non-responders (blue), asymptomatic (green), and responders (black). The algorithm did not define a separate group of six adverse responders as distinguished by the manual method. However, five of these subjects clustered together in the asymptomatic group, and one was placed among the non-responders group (blue) by the algorithm. The Ward hierarchical clustering algorithm also classified three responder subjects (black) among the asymptomatic (green), which is different from the manual classification. Note that these four subjects differently classified by the manual and computational methods all lie very close to the previously defined arbitrary cut-off (Fig. 2D).

### The virtual clinical trial identified potential biomarkers

**Figure 3:**
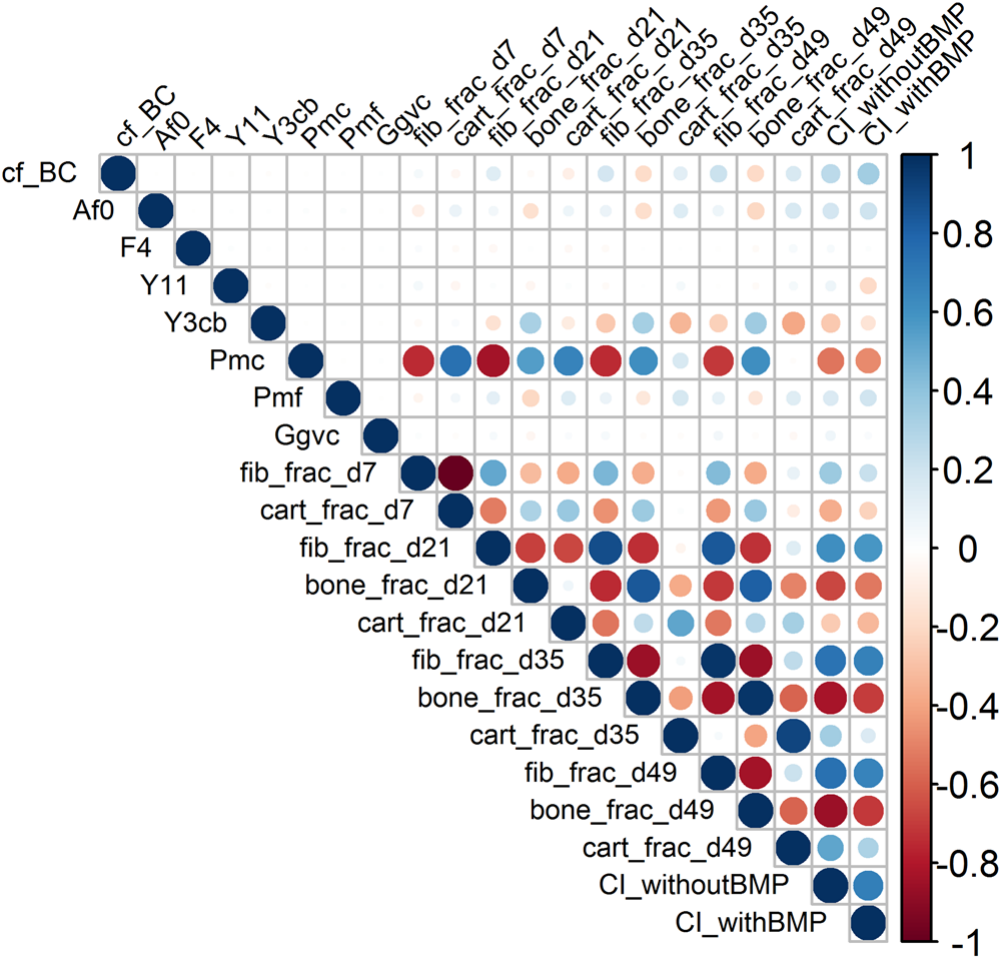
Graphical display of the Spearman correlation matrix. Note that the NF1 parameters (cf_BC, Af0, F4, *Y*_*11*_, Y3cb, Pmc, Pmf, Gfvc) are uncorrelated. Fib = fibrous tissue, cart = cartilage, frac = fraction, dx = day x.

Next, we investigated whether the manually defined subject classes have distinctive characteristics that can be used as potential biomarkers to predict the efficacy of BMP treatment. We focused here on the difference between the responders (black) and the non-responders (blue), as these subjects can benefit most from BMP treatment. We first calculated the Spearman correlation matrix of the dataset including the 8 NF1 parameters as well as fibrous, cartilage, and bone tissue fractions at different time points to check for highly correlated (redundant) attributes (Fig. 3). Importantly, the results showed that the NF1 parameters were uncorrelated. As expected, the CI value was negatively correlated with the bone tissue fraction and positively correlated with the fibrous tissue fraction. Similarly, some NF1 parameters were correlated with certain tissue fraction outcomes (e.g., *A*_*f0*_, which describes the rate of fibroblastic proliferation (see *Methods*), with the amount of fibrous tissue), and the tissue fractions were also not independent.

Using the 8 NF1-related parameter values in data-driven modeling, we determined which parameter was most important in predicting stratification between paired subject groups (Table 1, ROC-curves can be found in the supplementary material). Note that not all comparisons are balanced, particularly for the adverse responders with only 6 subjects. Across all paired groups, *P*_*mc*_, *Y*_*11*_, and *Y*_*3cb*_ are important predictors (Table 1, Supplementary Figure 1), with mean accuracy values >0.72. *P*_*mc*_ represents the rate of cartilage formation, *Y*_*11*_ signifies osteogenic differentiation, and *Y*_*3cb*_ denotes endochondral ossification (see *Methods*). Importantly, when similar analyses are performed on scrambled data, a mean accuracy of 0.48 was obtained, and no clear predictors were found (Supplementary Figure S1D–F). These data independently validated the accurate stratification of the subject groups.

**Table 1:** The NF1-related parameters with greatest predictive power to differentiate the paired subject groups in the virtual clinical trial^a^.

SUBJECT GROUP	Responders (*n* = 47)	Adverse responders (*n* = 6)	Non-responders (*n* = 47)	Asymptomatic (*n* = 100)
Responders		*Y*_*11*_ (0.91 ± 0.05)	*P*_*mc*_ (0.72 ± 0.08)	*P*_*mc*_, *Y*_*3cb*_ (0.86 ± 0.05)
Adverse responders			*Y*_*11*_, *P*_*mc*_ (0.92 ± 0.05)	*Y*_*11*_ (0.96 ± 0.02)
Non-responders				*P*_*mc*_ (0.96 ± 0.03)
Asymptomatic				

^a^Predictors are as defined in the *Methods* and based on mean arbitrary units >50 with a random forest, binary classification model. Mean accuracy ± standard deviation is given in parentheses and were measured across 100 trained random forest models.

**Figure 4:**
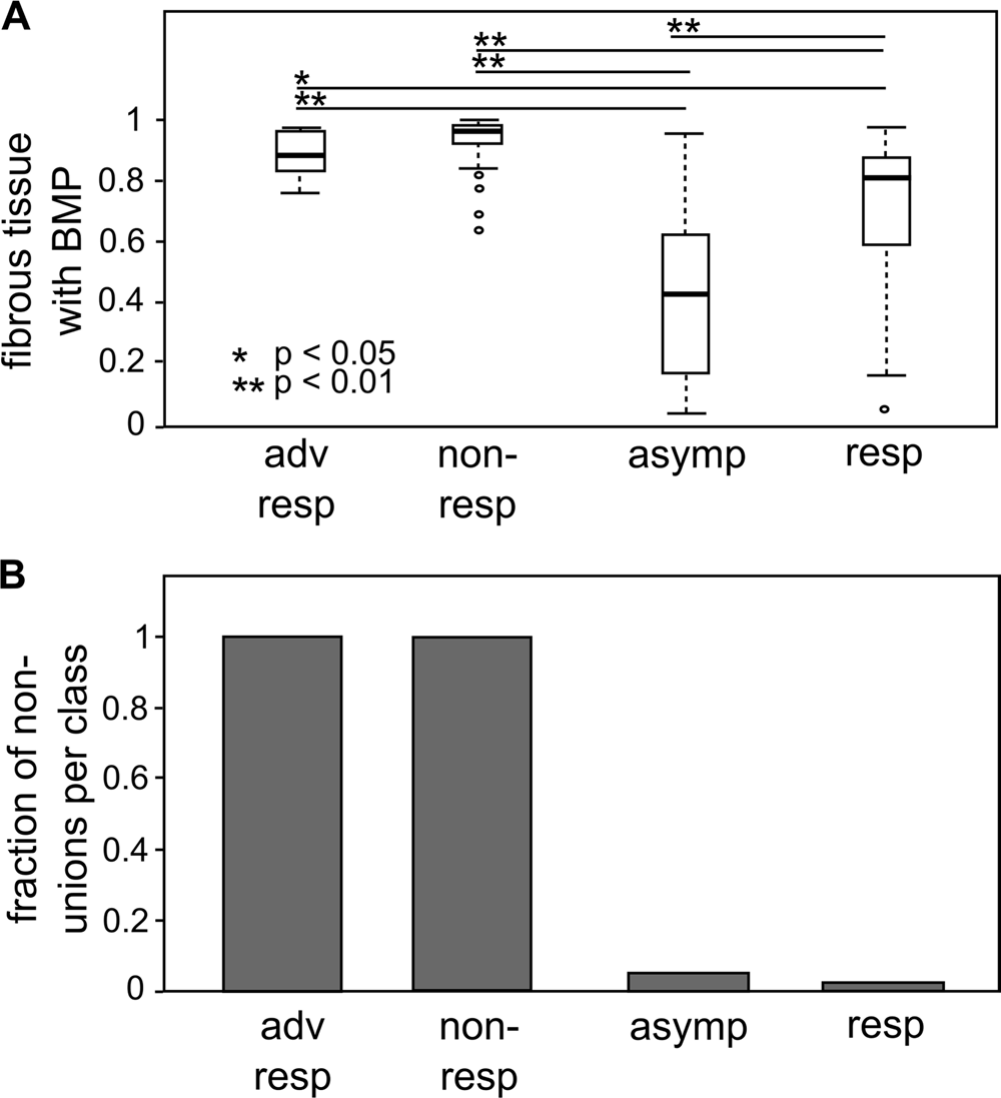
(**A**) Box plot summarizing the amount of fibrous tissue at day 49 after treatment by subject class. (**B**) Bar plot summarizing the fraction of non-unions per subject class. Adv resp = adverse responders (*n* = 6); non-resp = non-responders (*n* = 47); asymp = asymptomatic (*n* = 100); resp = responders (*n* = 47).

In order to analyze the data on CI in more detail, we looked at the different components constituting the CI value, e.g., the amount of fibrous tissue after 49 days and the amount of non-unions (Fig. 4A). Interestingly, the box plots show that there was a significant difference in the amount of fibrous tissue present at day 49 after treatment for the different subject classes, with significantly less fibrous tissue present in the responding subjects. Moreover, the fraction of non-unions is also lower in the responding subjects (Fig. 4B).

## Discussion

The clinical literature reports conflicting evidence about the effectiveness of BMP in treating CPT^13,14,17,18^. It is difficult to compare these clinical studies and draw solid conclusions for various reasons. First the surgical procedures and fixation methods are very heterogeneous, varying between Ilizarov fixation^17^, intramedullary fixation^14^, autologous grafting^18^, Masquelet technique^13^, or a combination thereof. Second, some clinical studies are performed with rhBMP-7^18^ whereas others use rhBMP-2^14^. Third, the delivery (e.g., mixed with collagen and allograft bone^18^ or the use of a collagen sponge alone^14^) and the dosage of the rhBMP (e.g., from 3.5^18^ to 12 mg^14^) varied between studies. Fourth, with CPT being an orphan disease, the few case studies with limited follow-up of refracture result in a small amount of patient-specific data. Finally, and most importantly, the treated CPT patients each have a specific case history with previous treatments, including multiple surgeries and rounds of BMP treatment, which makes conclusions hazardous.

Since virtual clinical trials alleviate some of the above mentioned problems, we combined in this study mechanistic modeling with data-driven modeling in an investigative *in silico* clinical trial to determine the (beneficial) effect of BMP treatment on fracture healing in NF1 subjects. A large *in silico* trial was designed, simulated and analyzed, based on a previously established computational model of murine bone regeneration. Notably, the computational model has been previously corroborated in various pathological conditions including small bone defects^19^, large bone defects^20,21^ and CPT in NF1 subjects^16^. Although the parameters and the *in silico* clinical results are related through mathematical equations, the set of partial differential equations is very extensive and has many non-linear interactions between the variables. As such it is impossible to intuitively predict how the parameter values will influence the *in silico* predictions, here the bone healing outcome. Therefore, in this study, we decided to generate an extensive data set *in silico* and mine it using machine learning approaches.

An important simulation outcome is the CI value, which reflects the degree of severity of CPT. We found that the CI value was significantly lower with BMP, compared to no treatment, for the 200 subjects in our *in silico* clinical trial (Fig. 2). Our computational model predicted a wide range (0.01–0.89) of CI values for both treated and untreated conditions, reminiscent of the substantial variation in severity of pseudarthrosis, which suggests that not all subjects benefit from treatment. The large range of predicted CI values for the non-treated subjects is remarkable since the parameter values of the eight factors were mostly varied in one direction with respect to the baseline value, thereby biasing the design to a CPT phenotype. By manual thresholding the CI values, four distinct subject groups were identified: adverse responders, responders, non-responders and asymptomatic. Unsupervised Ward hierarchical clustering confirmed the existence of all classes except the adverse responders, although these subjects also clustered together. The existence of four distinct subject groups may explain the heterogeneity in the reported clinical outcomes mentioned above, although further research is necessary to confirm this subject stratification and find biomarkers thereof, in particular for the small subpopulation of subjects predicted to negatively react to the BMP treatment.

It is also noteworthy that the *in silico* responders (*n* = 47) have a wide range in the amount of fibrous tissue at day 49 (Fig. 4A). The presence of fibrous tissue in the regenerating tissue may correlate to inferior mechanical properties and a higher risk for refracture. As such, the computational results seem to confirm the clinical observation that BMP induces healing but with a substantial refracture risk although this biomechanical hypothesis should be investigated further.

In addition to subject stratification, the data-driven modeling techniques allowed us to identify three NF1 parameters that can accurately predict stratification among paired subject groups (Table 1). With further validation, these parameters might form the basis of biomarkers for successful BMP treatment. The rate of cartilage formation (*P*_*mc*_) is a distinguishing factor between the non-responders and the responders. This result corresponds to the findings of a previous computational study^16^ where it was shown that *P*_*mc*_ is a critical determinant for the CI value due its important role in the endochondral ossification pathway. Interestingly, *Y*_*3cb*_, a factor representing endochondral ossification, was also identified as a predictive NF1 parameter. It should be noted that *P*_*mc*_ and *Y*_*3cb*_ are not correlated.

While this study focused specifically on the design, execution and analysis of an investigative *in silico* clinical trial for CPT, a pediatric orphan disease, others have used *in silico* clinical trials to investigate different clinical challenges. Clermont *et al*. constructed a cohort of 1000 virtual trial subjects to evaluate immunomodulatory strategies for severe sepsis^22^ and Qasim *et al*. have developed 100 individualized finite element models to predict the risk of hip fractures in osteoporosis^23^. Besides refining and partially replacing animals or humans in a clinical trial, *in silico* models can also generate virtual patients with particular characteristics to augment clinical trials, which is particularly relevant for trials in children or rare diseases^24^.

In addition to their value in studying clinical problems, *in silico* clinical trials offer huge potential in the design-discovery phase of biomedical product development. These virtual studies can identify biomarkers, optimize the dosage and duration of therapeutic interventions and patient stratification as well as perform risk and safety assessments required for regulatory applications^10^. For example, Martelli *et al*. used finite element models to perform an extensive risk analysis and robustness assessment of novel epiphyseal hip implants^25^. Similarly, Wilkoff *et al*. successfully developed a comprehensive modeling framework to provide a robust safety evaluation of the lead electrode heating hazard when patients with implanted pacemakers and defibrillators are scanned in a magnetic resonance imaging environment^26^. Kovatchev *et al*. have established an *in silico* metabolic model to test the safety and limitations of closed-loop control algorithms that link subcutaneous glucose sensors to insulin pumps in an artificial pancreas^27^. Importantly, the *in silico* metabolic model has been shown to adequately represent glucose fluctuations in type 1 diabetes and has been accepted by the Food and Drug Administration as a substitute to animal trials in the preclinical testing of closed-loop control algorithms. Notably, because of the increasing interest in the use of *in silico* models, frameworks and guidelines are being developed to thoroughly investigate the reliability of *in silico* model predictions for regulatory applications^28^ and *in silico* clinical trials^6,29^.

Although the *in silico* clinical trial presented here offers much insight into treatment of CPT, we recognize several limitations. Firstly, the *in silico* clinical trial considers a cohort of (only) 200 murine subjects in order to balance between statistical accuracy and computational feasibility (one simulation requires on average 48 hours computing time on one node of a high performance cluster). The virtual CPT subjects were based on a murine model of bone regeneration, including a generic murine-based geometry, due to the lack of experimental human data and to reduce computational demands. *In silico* simulations have shown, however, that increasing the fracture geometry to human dimensions will proportionally increase the fracture healing time (unpublished data). Secondly, the virtual subjects were uniformly sampled across the parameter space which resulted in a large range of phenotypes and inherent variability in the data. From the additional data-driven analyses, four subject groups were identified but these were not similar in size. Moreover, the group size of the adverse responders was too small to draw any statistically significant conclusions. In the future, advanced algorithms, such as bootstrap aggregating, could be used to tackle the issues related to unequally sized clusters. A larger cohort of subjects could also be simulated, with an improved sampling of the group of adverse responders as well as a more refined definition of the manually identified groups. Thirdly, the degree of severity of CPT was assessed by the CI, previously defined by three phenotypic criteria: the absence of cortical bridging of the defect, the amount of fibrous tissue and the amount of fibroblasts^16^. The results of this clinical trial should be interpreted in light of this definition, which puts a lot of importance on the presence of fibroblasts and fibrous tissue.

Nevertheless, the combined mechanistic and data-driven modeling approach allows us to successfully perform and analyze an investigative *in silico* clinical trial, including the identification of subject subpopulations. An obvious next step would be to extensively characterize the real patient-specific parameter distributions and the corresponding distribution of clinical CI values and compare those with the simulated cohort. However, due to the particular nature of the disease (orphan, pediatric), it will be extremely difficult to obtain these distributions for a sufficiently high number of patients. As an intermediate proxy, we intend to characterize a number of *in vitro* properties such as trilineage differentiation capacity of NF1-haplodeficient cells and NF1-null cells from various donors. Another important step towards a clinical translation of these results is the *in vivo* validation of the *in silico* predictions in order to find out whether these subject subpopulations truly exist. Moreover, in case of discrepancies between the predicted and measured *in vivo* outcomes, the additional *in vivo* experimentation would allow for the identification of any shortcomings of the computational model, such as particular model simplifications (see ref.^16^ for a more elaborate discussion on model simplifications).

In summary, *in silico* models and virtual clinical trials are increasingly being explored to tackle the challenges associated with pediatric diseases and/or orphan diseases. This study demonstrates how mechanistic and data-driven modeling are useful tools to simulate and mine data from *in silico* clinical trials and stratify subject populations to improve current treatment strategies. This robust modeling approach can also be employed for biomarker identification, optimization of the dosage or of the duration of the proposed intervention. Moreover, this study shows the vast potential of *in silico* medicine technologies as well as their implications for other orphan diseases.

## Materials and Methods

### Design of the *in silico* clinical trial

The set of 200 virtual subjects was generated from a previously established multiscale computational model of murine bone regeneration, which has been corroborated for small bone defects^19^, large bone defects^20^, and CPT in NF1 subjects^16^. Although the exact etiology of CPT is still unknown, 40–80% of CPT patients are carriers of a mutation in the *NF1* gene. Also, it has been postulated that double inactivation of the *NF1* gene is necessary for the development of pseudarthrosis^30,31^, and that the relative proportion of *NF1*-haplodeficient cells and *NF1*-null cells in the pseudarthrosis region will determine the healing outcome. To examine the effect of the *NF1* mutation on bone-fracture healing, the parameter values of the factors describing the aberrant cellular behavior of *NF1*-haplodeficient and *NF1-*null cells were varied across a wide range in the computational model (Table 2) to account for the variable proportion of *NF1*-haplodeficient and *NF1-*null cells in the pseudarthrosis region. In particular, a larger deviation of the parameter values from the normal case represents a higher proportion of *NF1*-null cells. We focused on the influence of eight factors described in the literature as contributors to the poor fracture-healing outcome in CPT patients: increased invasion of fibrous lesion cells (*c*_*f*,*BC*_)^12^, increased fibroblastic proliferation (*A*_*f*0_)^12^, increased fibroblastic differentiation (*F*_4_)^32^, reduced osteogenic differentiation (*Y*_*11*_)^12,33^, reduced endochondral ossification (*Y*_3,*cb*_)^12^, reduced cartilage formation (*P*_*mc*_)^12^, increased fibrous tissue formation (*P*_*mf*_)^32^, and increased angiogenic growth factor production (*G*_*gvc*_)^12,34^. Importantly, the computational model focuses on the cellular and biochemical aspects of CPT and implicitly assumes that the fracture is sufficiently stabilized to allow bone formation. Hence, mechanoregulatory stimuli were not considered in the computational model.

**Table 2:** Parameter values of the factors describing the aberrant cellular behavior of *NF1*-mutated cells.

Factor	Symbol	Normal case	NF1 range
Invasion time fibroblasts	*c*_*f*,*BC*_	3	0–50
Fibroblastic proliferation	*A*_*f*0_	0.1	0.1–10
Fibroblastic differentiation	*F*_4_	0.01	0.01–1
Osteogenic differentiation	*Y*_*11*_	20	0–20
Endochondral ossification	*Y*_3,*cb*_	1000	0–1000
Cartilage formation	*P*_*mc*_	0.2	0–0.2
Fibrous tissue formation	*P*_*mf*_	0.2	0.2–10
Angiogenic growth factor production	*G*_*gvc*_	10^3^	10^3^–10^5^

The *in silico* clinical trial consisted of 200 virtual subjects for which the healing process was simulated both with BMP treatment and without treatment. The 200 virtual subjects were generated using the “Design of Experiments” (DOE) tool of JMP (SAS Institute Inc., Cary, North Carolina, USA)^35,36^. DOE (or experimental design) is a statistical method that generates an array of combinations of different parameter values within a predefined parameter space. A uniform design was chosen since the computational model of bone regeneration is highly non-linear. Uniform designs also cope well with the addition and removal of runs. The parameter space (Table 2, NF1 range) was determined by varying the parameter values of the eight factors mostly in one direction with respect to the normal case, in this way biasing the DOE design to a CPT phenotype. As such, each virtual CPT subject had a unique combination of eight parameter values, representing their intrinsic cellular and biochemical properties. Please note that the resection of the fibrous hamartoma, consisting of *NF1*-haploinsufficient and *NF1-*null cells, is not captured in the *in silico* model, or in other words, it models the worst-case scenario where *NF1-*null cells are still present in the fracture callus. The computer simulations were performed with these parameter combinations, utilizing high-performance computational resources provided by the VSC (Flemish Supercomputer Center).

BMP treatment was modeled using one generic osteochondrogenic growth factor (*g*_*bc*_), which represents the effect of multiple growth factors present in the fracture callus and released from the BMP sponge as a clinical treatment. The influence of this generic growth factor on differentiation is either chondrogenic or osteogenic depending on the local oxygen tension. To model the gradual release of BMP from a BMP sponge, implanted directly after the occurrence of the fracture, a time-dependent, periosteal boundary condition was simulated (Eq. 1):

1 $${g}_{bc,BC}={g}_{bc,BC}^{\ast }\,.{e}^{-t/\tau }$$

with τ the time constant of the exponential decay, chosen to be equal to 10 days^37,38^. ${g}_{bc,BC}^{\ast }$ is equal to the standard value of the osteochondrogenic boundary condition (see supplementary material).

The degree of severity of CPT is assessed by the complication index (CI, *γ*_7_), which was previously defined in ref.^16^ as a linear combination of the three most prevalent symptoms of CPT (a non-union (*γ*_6_), harmatoma (*γ*_4_), and presence of fibroblasts (*γ*_5_)) (Eq. 2):

2 $${\gamma }_{7}=\frac{{\gamma }_{4}+{\gamma }_{5}+{\gamma }_{6}}{3}$$

A parameter combination yielding a small CI value reflects a small degree of severity of CPT, or in other words, the fracture healing proceeds fairly normally. Conversely, a parameter combination resulting in a large CI value represents severely impaired fracture healing and is reminiscent of the CPT phenotype.

### Statistical analysis

For the statistical analysis of the CI values with and without BMP treatment, a two-sided, paired t-test was performed. To classify subjects based on their CI values with and without BMP treatment, we used the Ward hierarchical clustering algorithm (ward.D2, Euclidean distance) from the “stats” package implemented in R (version 3.3.1)^39^. To determine the prediction accuracy of the classification and the most important predictors, we created a machine-learning model with the “caret”^31^ package employing the random classification algorithm from the “randomForest” package in R^40^. The random forest model was trained on 75% of the data with 10-fold cross-validation^41^. The accuracy was assessed on the remaining 25% of the data. To get sufficient precision, the random forest model was trained 100 times with random splitting of data in training and testing data set every time. The same analyses were also performed on scrambled data, obtained with the randomization algorithm from the “picante” package^42^. The script used to perform the statistical analyses is provided in the supplementary material.

## Electronic supplementary material

Supplementary Material

Dataset 1
