# Supplementary material for: In silico clinical trials for pediatric orphan diseases
Source: Sci Rep. 2018 Feb 6;8:2465. doi: 10.1038/s41598-018-20737-y (PMC5802824; doi:10.1038/s41598-018-20737-y)
Supplement: Supplementary file 1 — Supplementary Material [file 41598_2018_20737_MOESM1_ESM.pdf]

In silico clinical trials for pediatric orphan diseases

A. Carlier[1,2,3], A. Vasilevich[3], M. Marechal[2,4], J. de Boer[3], L. Geris[1,2]*

[1] Biomechanics Section, KU Leuven, Celestijnenlaan 300 C, PB 2419, 3000 Leuven, Belgium and Biomechanics Research Unit, University of Liège, Chemin des Chevreuils 1 – BAT 52/3, 4000 Liège 1, Belgium.
[2] Prometheus, Division of Skeletal Tissue Engineering, KU Leuven, O&N 1, Herestraat 49, PB 813, 3000 Leuven, Belgium.
[3] MERLN Institute for Technology-Inspired Regenerative Medicine, Maastricht University, Universiteitssingel 40, 6229 ER, Maastricht, The Netherlands.

[4] Skeletal Biology and Engineering Research Center, KU Leuven, O&N 1, Herestraat 49, PB 813, 3000 Leuven, Belgium.

*Corresponding author. Please address to:
Biomechanics Research Unit
GIGA In Silico Medicine
Université de Liège
Quartier Polytechnique, Allée de la découverte 13A (B52/3)
B-4000 Liège, Belgium
tel +32 43 66 95 87
fax +32 43 66 95 05
e-mail: liesbet.geris@ulg.ac.be

Keywords: machine learning, neurofibromatosis, simulation, virtual patient cohort, mechanistic modeling

Supplementary material

Supplementary material 2
1. Description of the standard mathematical model 3
1.1 Continuous description of fracture healing (taxis-reaction-diffusion type PDEs) 3
1.2 Discrete description of angiogenesis (agent-based) 8
1.3 Scaling parameters and parameter values 12
1.4 Boundary and initial conditions 15
1.5 Implementation 17
2. Description of the NF1 model 19
3. Additional information 22
4. Supplementary R-code 26
5. References 73

1. Description of the standard mathematical model

1.1 Continuous description of fracture healing (taxis-reaction-diffusion type PDEs)

The following equations describe the spatiotemporal evolution of the densities of mesenchymal stem cells (c_m), fibroblasts (c_f), chondrocytes (c_c), osteoblasts (c_b), fibrous tissue (m_f), cartilage (m_c), bone (m_b) and concentrations of osteochondrogenic (g_{bc}) and vascular growth factors (g_v) and oxygen tension (n):

$$\frac{\partial c_m}{\partial t} = \nabla\left[\overbrace{D_m \nabla c_m}^{diffusion} - \overbrace{C_{m_{CT}} c_m \nabla(g_{bc}+g_v)}^{chemotaxis} - \overbrace{C_{m_{HT}} c_m \nabla m}^{haptotaxis}\right] + \overbrace{A_m c_m\left[1-\alpha_m c_m\right]}^{proliferation} \underbrace{-\ F_1 c_m}_{\substack{osteogenic\\differentation}} \underbrace{-\ F_2 c_m}_{\substack{chondrogenic\\differentiation}} - \overbrace{F_4 c_m}^{\substack{fibroblastic\\differentiation}} - \overbrace{F_7 c_m}^{apoptosis} \qquad (S.1)$$

$$\frac{\partial c_f}{\partial t} = \nabla\left[\overbrace{D_f \nabla c_f}^{diffusion} - \overbrace{C_f c_f \nabla g_{bc}}^{chemotaxis}\right] + \overbrace{A_f c_f\left[1-\alpha_f c_f\right]}^{proliferation} + \underbrace{F_4 c_m}_{\substack{fibroblastic\\differentiation}} - \overbrace{F_3 d_f c_f}^{\substack{endochondral\\ossification}} - \underbrace{F_8 c_f}_{apoptosis} \qquad (S.2)$$

$$\frac{\partial c_c}{\partial t} = \overbrace{A_c c_c\left[1-\alpha_c c_c\right]}^{proliferation} + \underbrace{F_2 c_m}_{\substack{chondrogenic\\differentiation}} - \underbrace{F_3 c_c}_{\substack{endochondral\\ossification}} - \underbrace{F_5 c_c}_{apoptosis} \qquad (S.3)$$

$$\frac{\partial c_b}{\partial t} = \overbrace{A_b c_b\left[1-\alpha_b c_b\right]}^{proliferation} + \underbrace{F_1 c_m}_{\substack{osteogenic\\differentiation}} + \underbrace{F_3 c_c}_{\substack{endochondral\\ossification}} - \underbrace{F_6 c_b}_{apoptosis} - \underbrace{d_b c_b}_{\substack{osteocytic\\differentiation}} \qquad (S.4)$$

$$\frac{\partial m_f}{\partial t} = \overbrace{P_{fs}\left(1-\kappa_f m_f\right)c_f}^{production} - \overbrace{Q_f m_f m_c c_b}^{resorption} \qquad (S.5)$$

$$\frac{\partial m_c}{\partial t} = \overbrace{P_{cs}\left(1-\kappa_c m_c\right)c_c}^{production} - \overbrace{Q_c m_c c_b}^{resorption} \qquad (S.6)$$

$$\frac{\partial m_b}{\partial t} = \overbrace{P_{bs}\left(1-\kappa_b m_b\right)c_b}^{production} \tag{S.7}$$

$$\frac{\partial g_{bc}}{\partial t} = \nabla\overbrace{\left[D_{gbc}\nabla g_{bc}\right]}^{diffusion} + \overbrace{E_{gb}c_b + E_{gc}c_c}^{production} - \overbrace{d_{gbc}g_{bc}}^{denaturation} \tag{S.8}$$

$$\frac{\partial g_v}{\partial t} = \nabla\overbrace{\left[D_{gv}\nabla g_v\right]}^{diffusion} + \overbrace{E_{gvb}c_b + E_{gvc}c_c}^{\substack{hypoxia-independent\\ production}} - g_v\left(\overbrace{d_{gv}}^{denaturation} + \overbrace{d_{gvc}c_v}^{\substack{cellular\\ uptake}}\right) + \overbrace{E_{hyp,b}c_b + E_{hyp,\mathrm{c}}c_c + E_{hyp,\mathrm{f}}c_f + E_{hyp,\mathrm{m}}c_m}^{\substack{hypoxia-dependent\\ production}} \tag{S.9}$$

$$\frac{\partial n}{\partial t} = \nabla\overbrace{\left[D_n\nabla n\right]}^{diffusion} + \overbrace{E_n c_v}^{production} - \overbrace{d_{n,b}c_b - d_{n,\mathrm{c}}c_c - d_{n,\mathrm{f}}c_f - d_{n,\mathrm{m}}c_m}^{\substack{cellular\\ consumption}} \tag{S.10}$$

where m (= m_f + m_c + m_b) represents the total tissue density. The migration of mesenchymal stem cells is a combination of random and directed motion [1]. The random motion was modelled as a haptokinetic process. Random motion is influenced by the total matrix density, defined as $m = m_f + m_c + m_b$, such that in the absence or abundance of extracellular matrix cells cease to move.

$$D_m = \frac{D_{hm}m}{K_{hm}^2 + m^2} \tag{S.11}$$

Chemotaxis was modelled using a receptor-kinetic form, giving a maximum chemotactic response at a particular growth factor concentration [2]. The chemotactic response of mesenchymal stem cells depends both on osteogenic and angiogenic growth factors.

$$C_{m_{CT}} = \frac{C_{kCTm}(g_{bc}+g_v)}{K_{kCTm}^2+(g_{bc}+g_v)^2}$$

$$C_f = \frac{C_{kf}\,g_{bc}}{K_{kf}^2+g_{bc}{}^2} \tag{S.12}$$

The haptotactic coefficient was taken from [3], based on a kinetic analysis of a model mechanism for the cell-surface-receptor-extracellular-ligand binding dynamics [4].

$$C_{m_{HT}} = \frac{C_{kHTm}}{\left(K_{kHTm}+m\right)^2} \tag{S.13}$$

The proliferation of stem cells, as well as the other three cell types, is modelled by a logistic growth function, whereby the proliferation rate depends on the surrounding matrix density and oxygen tension [3,5].

$$A_i = \frac{A_{i0}m}{K_i^2+m^2}\cdot\frac{A_{in}n}{K_{in}^3+n^3} \tag{S.14}$$

with $i = m$ for MSCs (c_m) and

$$A_i = \frac{A_{i0}m}{K_i^2+m^2}\cdot\frac{A_{in}n}{K_{in}^2+n^2} \tag{S.15}$$

with $i = f$ for fibroblasts (c_f) and

$$A_i = \frac{A_{i0}m}{K_i^2+m^2}\cdot\frac{A_{in}n}{K_{in}^6+n^6} \tag{S.16}$$

with $i = c, b$ for chondrocytes (c_c) and osteoblasts (c_b). The differentiation of MSCs towards osteoblasts is mediated by the presence of osteochondrogenic growth factors [6,7] and oxygen tension [8]. For high chemical concentrations, a saturation effect was modelled to take place [9].

$$F_1 = \frac{Y_{11}\cdot g_{bc}}{H_{11}+g_{bc}}\cdot\frac{Y_{12}.n^6}{I_v^6+n^6} \tag{S.17}$$

A similar function was used to model the differentiation towards chondrocytes:

$$F_2 = \frac{Y_2 g_{bc}}{H_2 + g_{bc}} \cdot \frac{Y_{2n} n}{K_{2n}^6 + n^6} \tag{S.18}$$

The following function describes the endochondral replacement of chondrocytes [10,11]:

$$F_3 = \frac{m_c^6}{B_{ec}^6 + m_c^6} \cdot \frac{Y_3 g_{bc}}{H_3 + g_{bc}} \cdot \frac{n^6}{B_v^6 + n^6} \tag{S.19}$$

The production of osteochondrogenic growth factors by chondrocytes occurs up to a certain saturation concentration, after which the production rate levels off. The production is also dependent on the matrix density:

$$E_{gc} = \frac{G_{gc} g_{bc}}{\left(H_{gc} + g_{bc}\right)} \cdot \frac{m}{\left(K_{gc}^3 + m^3\right)} \tag{S.20}$$

The production of osteochondrogenic growth factor by osteoblasts was modelled in a similar way, except that the osteochondrogenic growth factor production rate is not limited by matrix density.

$$E_{gb} = \frac{G_{gb} g_{bc}}{H_{gb} + g_{bc}} \tag{S.21}$$

The hypoxia-independent production of angiogenic growth factors occurs by osteoblasts and hypertrophic chondrocytes. The production rate saturates for high angiogenic growth factor concentrations, leading to the following function for the production rates by osteoblasts E_{gvb} and hypertrophic chondrocytes E_{gvc}:

$$E_{gvb} = \frac{G_{gvb} H_{gv}^6}{\left(H_{gv}^6 + g_v^6\right)} \tag{S.22}$$

$$E_{gvc} = \frac{G_{gvc} H_{gv}^6}{\left(H_{gv}^6 + g_v^6\right)} \cdot \frac{m_c^6}{\left(B_{ec}^6 + m_c^6\right)} \quad \text{(S.23)}$$

All the cell types produce angiogenic growth factors in a hypoxia-dependent manner, molled as follows:

$$E_{hyp,i} = \frac{Q_{hyp,i} K^6{}_{hyp,i}}{K^6{}_{hyp,i} + n^6} \quad \text{(S.24)}$$

with *i = m, f, b, c* for MSCs, fibroblasts, osteoblasts and chondrocytes respectively. The release of oxygen by the blood vessels is modeled according to:

$$E_n = \frac{G_n H_n^6}{H_n^6 + n^6} \quad \text{(S.25)}$$

Similar to Demol et al., the cellular consumption of oxygen was described using a Michaelis-Menten kinetic law [12]:

$$d_{ni} = \frac{Q_{ni} n}{K_{ni} + n} \quad \text{(S.26)}$$

with *i = m, f, b, c* for MSCs, fibroblasts, osteoblasts and chondrocytes respectively. In pathologically low oxygen environments, cellular metabolism is compromised resulting in cell death.

$$F_5 = F_{5,1} + F_{5,2} = \frac{A_{5n1} H_{5n1}{}^6}{H_{5n1}{}^6 + n^6} + \frac{A_{5n2} n^6}{H_{5n2}{}^6 + n^6} \quad \text{(S.27)}$$

for chondrocytes and

$$F_{6,7,8} = \frac{A_{in} H_{in}{}^6}{H_{in}{}^6 + n^6} \quad \text{(S.28)}$$

with *i = 6, 7, 8* for osteoblasts, MSCs and fibroblasts respectively. Remark that the death term of chondrocytes consists of two contributions: increased cell death in very low (0.5% oxygen tension) and high oxygen tensions (11% oxygen tension) [13] whereas the other cell types only have one term, i.e. they can survive in well oxygenated environments (bearing in mind that in the model simulations oxygen tension is not exceeding 12%).

1.2 Discrete description of angiogenesis (agent-based)

The discrete variable c_v represents the blood vessel network, which is implemented on a lattice. When a grid volume contains an endothelial cell this variable is set to 1, otherwise $c_v = 0$. The vessel diameter is defined by the grid resolution and is always one endothelial cell wide, although the movement of the tip cell is grid independent as explained below. Every endothelial cell ($c_v = 1$) has unique intracellular protein levels, which control the behavior of that specific cell. The intracellular module is adapted from the agent-based model of Bentley et al. [14] and consists of the following variables: the level of VEGFR-2 (*V*), Notch1 (*N*), Dll4 (*D*), active VEGFR-2 (*V′*), active Notch1 (*N′*), effective active VEGFR-2 (*V″*), effective active Notch1 (*N″*) and the amount of actin (*A*). The effective active levels (*V″* and *N″*) include the time delay of translocation to the nucleus, thereby representing the levels at the nucleus, influencing gene expression. The amount of actin (*A*) refers to the polymerized actin levels (F-actin) inside the cell. In particular, it is associated to actin used for filopodia formation, owing to its importance for tip cell migration. As such, an increase in actin levels can be interpreted as filopodia extension, while a decrease as filopodia retraction. Other intracellular signaling pathways that involve actin, such as energy metabolism [15,16], are not considered.

The activation of the VEGFR-2 receptor, described by V', is given by:

$$V_t^{'} = \frac{V_{\sin k} . V_{t-\delta t} . M_{tot}}{V_{\max}} . g_v(t) \tag{S.29}$$

where the constant V_{sink} represents the amount of VEGFR-1 receptors that act as a sink (decoy receptor) by removing VEGF from the system, t represents the time and δt the time step of the inner loop, V_{max} represents the maximal amount of VEGFR-2 receptors, g_v is the local VEGF concentration (at the tissue level) and M_{tot} is the total number of membrane agents (constant for all ECs). The level of activated VEGFR-2 remains in a range going from 0 to V. When the VEGFR-2 receptors are activated above a certain threshold ($V'*$), the actin levels of the endothelial cell are incremented in a constant manner (ΔA). As mentioned earlier, this represents the extension of filopodia by the endothelial cell, which is shown to be regulated downstream of VEGFR-2 [17]. If the endothelial cell fails to extend its filopodia for a certain amount of time D_3, the filopodia retract which is mathematically translated into a reduction of the actin levels in a constant manner (-10.ΔA). The actin level remains in a range between 0 and A_{max}. The amount of Notch1 is considered to be constant in every EC, representing a balance between the rate of degradation and expression. At the same time, initial Notch activity levels are assumed to be zero and in the model Notch activity can only be increased through binding with Dll4 from neighboring ECs. The model therefore neglects the role of Notch in EC quiescence and the fact that high Notch activity levels have been measured in quiescent ECs [18–20]. Instead, it only focuses on the role of Dll4-Notch in tip cell selection. The number of activated Notch receptors (N') will be equal to the total amount of Dll4 available (with an upper bound, given by the total number of Notch receptors N). The amount of Dll4 in the environment of an EC is the sum of the amount of Dll4 at the junctions with its neighboring ECs, whereby

every cell is assumed to distribute Dll4 uniformly across its cell-cell junctions. After a delay of D_1 for V' and D_2 for N' the active VEGFR-2 and Notch levels become the effective active levels (V'' and N'') representing the levels at the nucleus, influencing gene expression. The delays were taken from Bentley et al. which were fitted to a somite clock Delta-Notch system [14,21]. Note that there is a delay between receptor activation and gene expression (transcription) but not between gene expression and protein synthesis (translation), which is a simplification of the model. The amount of Dll4 is modeled in the following way:

$$D_t = D_{t-\delta t} + V''_{t-\delta t}.\delta - N'_{t-\delta t,neighbours} \qquad \text{(S.30)}$$

$D_{t-\delta t}$ represents the previous amount of Dll4, δ the change in Dll4 expression due to the activation of the VEGFR-2 receptor [17,22] and $N'_{t-\delta t,neighbours}$ is the amount of Dll4 that is removed from the environment due to the activation of Notch-receptors on neighboring ECs. If-conditions are used to ensure that the Dll4 level remains in a range between 0 and D_{max}. When Notch signaling is activated in a cell, the amount of VEGFR-2 receptors is down-regulated, suppressing the tip cell phenotype as follows [17,23]:

$$V_t = V_{\max} - N''_{t-\delta t}.\sigma \qquad \text{(S.31)}$$

V_{max} represents the maximal amount of VEGFR-2 receptors and σ models the VEGFR-2 expression change due to Notch1 activation. If-conditions are used to ensure that the VEGFR-2 level remains in a range going from V_{min} to V_{max}. Since the amount of VEGFR-2 (V) at the previous timestep ($V_{t-\delta t}$) is not present in Equation S.31, the amount of VEGFR-2 is continuously in equilibrium with the amount of effective active Notch1 ($N''_{t-\delta t}$). Equation S.31 implies that in quiescent cells the number of VEGFR-2 receptors will be maximal, owing to the

absence of any Notch activity. As mentioned earlier, the model neglects the role of Notch activity in quiescence and the fact that it will lead to reduced VEGFR-2 levels in quiescent ECs [18–20].

The evolution of the vascular network is determined by tip cell movement, sprouting and anastomosis [24,25], outlined below. The model computes the movement of every tip cell in a lattice-free manner. The cells that trail behind this tip are subsequently considered to be endothelial cells. Consequently, $\frac{d\overrightarrow{x_{tip}}}{dt} = v_{tip}\frac{\overrightarrow{u_{tip}}}{\left\|\overrightarrow{u_{tip}}\right\|_2}$ although the movement of a tip cell is grid independent, the vessel diameter is defined by the grid resolution due to the projection of the blood vessels on the grid. The movement of the tip cells is determined by their direction and speed, which is described by the tip cell velocity equations:

(S.32)

where $\overrightarrow{x_{tip}}$ represents the position, v_{tip} the speed and $\overrightarrow{u_{tip}}$ the direction of movement of the tip cell. The tip cell speed depends on the active VEGFR-2 concentration, meaning that both the surrounding VEGF concentration as well as the amount of VEGF-receptors influences the behavior of the tip cell [17,26]. Below a threshold activation level (*V′**) the tip cells do not migrate, above this, the tip cell velocity increases with *V′* up to a maximal speed of $v_{tip}^{\max}$. This translates into the following equation, where a third order polynomial was used to ensure a smooth threshold [24]:

$$v_{tip} = \begin{cases} 0 & if \quad V' < V'^* \\ v_{tip}^{\max}\dfrac{V'^3}{V'^3 + V'^{*3}} & if \quad V' \geq V'^* \end{cases} \qquad (S.33)$$

The direction of movement is influenced by chemotactic and haptotactic signals and is modeled in the same way as Peiffer et al. [24]. The tip cell phenotype is induced (formation of a new branch) or maintained (in already existing tip cells) if the following requirements are satisfied:

$$\begin{aligned} &V > \frac{V_{\max}}{2} \\ &A > A^* \end{aligned} \tag{S.34}$$

This criterion means that the endothelial cell must have enough VEGFR-2 and filopodia (polymerized actin) to sense the environment and direct the trailing branch towards the source of VEGF. When a tip cell encounters another blood vessel anastomosis takes place, after which the EC loses its tip cell phenotype.

1.3 Scaling parameters and parameter values

The following **scaling factors** were chosen for the non-dimensionalisation of the continuous variables:

$$\tilde{t} = \frac{t}{T}, \quad \tilde{x}_1 = \frac{x_1}{L}, \quad \tilde{x}_2 = \frac{x_2}{L}, \quad \tilde{x}_3 = \frac{x_3}{L}, \quad \tilde{c}_m = \frac{c_m}{c_0}, \quad \tilde{c}_f = \frac{c_f}{c_0},$$

$$\tilde{c}_c = \frac{c_c}{c_0}, \quad \tilde{c}_b = \frac{c_b}{c_0}, \quad \tilde{m}_f = \frac{m_f}{m_0}, \quad \tilde{m}_c = \frac{m_c}{m_0},$$

$$\tilde{m}_b = \frac{m_b}{m_0}, \quad \tilde{g}_{bc} = \frac{g_{bc}}{g_0}, \quad \tilde{g}_v = \frac{g_v}{g_0}, \quad \tilde{n} = \frac{n}{n_0}$$

Typical time and length scales for fracture healing in rodent studies are T = 1 day and L = 3.5 mm [27]. A representative concentration of the collagen content in the tissues under investigation is m_0 = 0.1 g/ml. Typical growth factor concentrations are in the order of magnitude of 10^{-9} M (mol/l) [28,29]. Taking into account the order of magnitude of the

molecular weight of the growth factors (100 kDa = 100 kg/mol), this results in a non-dimensionalisation value of g_0 = 100 ng/ml. Based on geometrical constraints, a typical value for cell density at the beginning of the healing process was taken to be: c_0 = 10^6 cells/ml [9]. A typical value for n_0 was chosen to be 100% (~1 mol/m^3). The parameter values were derived from literature where possible and estimated when no relevant data was available. We refer to Geris et al. [30] for a detailed description of the parameter derivation and estimation. The parameters were **non-dimensionalised** as follows (tildes referring to non-dimensionalised values):

$$\tilde{D}_{hm} = \frac{D_{hm}T}{L^2 m_0}, \quad \tilde{K}_{hm} = \frac{K_{hm}}{m_0}, \quad \tilde{C}_{kCTm} = \frac{C_{kCTm}T}{L^2}, \quad \tilde{K}_{kCTm} = \frac{K_{kCTm}}{g_0}, \quad \tilde{C}_{kHTm} = \frac{C_{kHTm}T}{L^2 m_0}, \quad \tilde{K}_{kHTm} = \frac{K_{kHTm}}{m_0}$$

$$\tilde{A}_{m0} = \frac{A_{m0}T}{m_0}, \quad \tilde{K}_m = \frac{K_m}{m_0}, \quad \tilde{A}_{mn} = \frac{A_{mn}}{n_0^2}, \quad \tilde{K}_{mn} = \frac{K_{mn}}{n_0}, \quad \tilde{Y}_{11} = Y_{11}T, \quad \tilde{H}_{11} = \frac{H_{11}}{g_0}, \quad \tilde{Y}_{12} = Y_{12}, \quad \tilde{I}_v = \frac{I_v}{n_0}$$

$$\tilde{Y}_2 = Y_2 T, \quad \tilde{H}_2 = \frac{H_2}{g_0}, \quad \tilde{Y}_{2n} = \frac{Y_{2n}}{n_0^5}, \quad \tilde{K}_{2n} = \frac{K_{2n}}{n_0}, \quad \tilde{F}_4 = F_4 T, \quad \tilde{\alpha}_m = \frac{\alpha_m}{c_0}, \quad \tilde{\alpha}_c = \frac{\alpha_c}{c_0}, \quad \tilde{\alpha}_b = \frac{\alpha_b}{c_0},$$

$$\tilde{\alpha}_f = \frac{\alpha_f}{c_0}, \quad \tilde{D}_f = \frac{D_f T}{L^2}, \quad \tilde{C}_{kf} = \frac{C_{kf}T}{L^2}, \quad \tilde{K}_{kf} = \frac{K_{kf}}{g_0}, \quad \tilde{A}_{f0} = \frac{A_{f0}T}{m_0}, \quad \tilde{K}_f = \frac{K_f}{m_0}, \quad \tilde{A}_{fn} = \frac{A_{fn}}{n_0},$$

$$\tilde{K}_{fn} = \frac{K_{fn}}{n_0}, \quad \tilde{B}_v = \frac{B_v}{n_0}, \quad \tilde{B}_{ec} = \frac{B_{ec}}{m_0}, \quad \tilde{A}_{5n1} = A_{5n1}T, \quad \tilde{H}_{5n1} = \frac{H_{5n1}}{n_0}, \quad \tilde{A}_{5n2} = A_{5n2}T, \quad \tilde{H}_{5n2} = \frac{H_{5n2}}{n_0},$$

$$\tilde{A}_{6n} = A_{6n}T, \quad \tilde{H}_{6n} = \frac{H_{6n}}{n_0}, \quad \tilde{A}_{7n} = A_{7n}T, \quad \tilde{H}_{7n} = \frac{H_{7n}}{n_0}, \quad \tilde{A}_{8n} = A_{8n}T, \quad \tilde{H}_{8n} = \frac{H_{8n}}{n_0}, \tilde{Y}_3 = Y_3 T, \quad \tilde{H}_3 = \frac{H_3}{g_0},$$

$$\tilde{A}_{c0} = \frac{A_{c0}T}{m_0}, \quad \tilde{K}_c = \frac{K_c}{m_0}, \quad \tilde{A}_{cn} = \frac{A_{cn}}{n_0^5}, \quad \tilde{K}_{cn} = \frac{K_{cn}}{n_0}, \quad \tilde{A}_{b0} = \frac{A_{b0}T}{m_0}, \quad \tilde{K}_b = \frac{K_b}{m_0}, \quad \tilde{A}_{bn} = A_{bn},$$

$$\tilde{K}_{bn} = \frac{K_{bn}}{n_0}, \quad \tilde{\kappa}_f = \frac{\kappa_f}{m_0}, \quad \tilde{\kappa}_c = \frac{\kappa_c}{m_0}, \quad \tilde{\kappa}_b = \frac{\kappa_b}{m_0}, \quad \tilde{d}_b = d_b T, \quad \tilde{P}_{fs} = \frac{P_{fs}Tc_0}{m_0}, \quad \tilde{Q}_f = Q_f m_0 c_0 T, \quad \tilde{d}_f = d_f T,$$

$$\tilde{P}_{cs} = \frac{P_{cs}Tc_0}{m_0}, \quad \tilde{Q}_c = Q_c c_0 T, \quad \tilde{P}_{bs} = \frac{P_{bs}Tc_0}{m_0}, \quad \tilde{D}_{gbc} = \frac{D_{gbc}T}{L^2}, \quad \tilde{G}_{gc} = \frac{G_{gc}Tc_0}{g_0 m_0^2}, \quad \tilde{H}_{gc} = \frac{H_{gc}}{g_0}, \quad \tilde{K}_{gc} = \frac{K_{gc}}{m_0},$$

$$\tilde{d}_{gbc} = d_{gbc}T, \quad \tilde{G}_{gb} = \frac{G_{gb}Tc_0}{g_0}, \quad \tilde{H}_{gb} = \frac{H_{gb}}{g_0}, \quad \tilde{D}_{gv} = \frac{D_{gv}T}{L^2}, \quad \tilde{G}_{gvb} = \frac{G_{gvb}Tc_0}{g_0 m_0^2}, \quad \tilde{H}_{gv} = \frac{H_{gv}}{g_0},$$

$$\tilde{G}_{gvc} = \frac{G_{gvc}Tc_0}{g_0 m_0^2}, \quad \tilde{d}_{gvc} = d_{gvc}T, \quad \tilde{d}_{gv} = d_{gv}T, \quad \tilde{Q}_{hyp,m} = \frac{Q_{hyp,m}Tc_0}{g_0}, \quad \tilde{K}_{hyp,m} = \frac{K_{hyp,m}}{n_0},$$

$$\tilde{Q}_{hyp,\mathrm{c}} = \frac{Q_{hyp,\mathrm{c}}Tc_0}{g_0}, \quad \tilde{K}_{hyp,\mathrm{c}} = \frac{K_{hyp,\mathrm{c}}}{n_0}, \quad \tilde{Q}_{hyp,\mathrm{b}} = \frac{Q_{hyp,\mathrm{b}}Tc_0}{g_0}, \quad \tilde{K}_{hyp,\mathrm{b}} = \frac{K_{hyp,\mathrm{b}}}{n_0}, \quad \tilde{Q}_{hyp,\mathrm{f}} = \frac{Q_{hyp,\mathrm{f}}Tc_0}{g_0},$$

$$\tilde{K}_{hyp,\mathrm{f}} = \frac{K_{hyp,\mathrm{f}}}{n_0}, \quad \tilde{D}_n = \frac{D_n T}{L^2}, \quad \tilde{G}_n = \frac{G_n Tc_0}{n_0}, \quad \tilde{H}_n = \frac{H_n}{n_0}, \quad \tilde{Q}_{n,m} = \frac{Q_{\mathrm{n,m}}Tc_0}{n_0}, \quad \tilde{K}_{n,m} = \frac{K_{\mathrm{n,m}}}{n_0},$$

$$\tilde{Q}_{n,\mathrm{c}} = \frac{Q_{\mathrm{n,c}}Tc_0}{n_0}, \quad \tilde{K}_{n,\mathrm{c}} = \frac{K_{\mathrm{n,c}}}{n_0}, \quad \tilde{Q}_{n,\mathrm{b}} = \frac{Q_{\mathrm{n,b}}Tc_0}{n_0}, \quad \tilde{K}_{n,\mathrm{b}} = \frac{K_{\mathrm{n,b}}}{n_0}, \quad \tilde{Q}_{n,\mathrm{f}} = \frac{Q_{\mathrm{n,f}}Tc_0}{n_0}, \quad \tilde{K}_{n,\mathrm{f}} = \frac{K_{\mathrm{n,f}}}{n_0}.$$

This resulted in the following set of **non-dimensional parameter values**:

$\tilde{D}_{hm} = 0.014, \quad \tilde{K}_{hm} = 0.25, \quad \tilde{C}_{kCTm} = 0.04, \quad \tilde{K}_{kCTm} = 0.1, \quad \tilde{C}_{kHTm} = 0.0034, \quad \tilde{K}_{kHTm} = 0.5,$
$\tilde{A}_{m0} = 0.4, \quad \tilde{K}_m = 0.1, \quad \tilde{A}_{mn} = 0.007, \quad \tilde{K}_{mn} = 0.05, \quad \tilde{Y}_{11} = 20.0, \quad \tilde{H}_{11} = 0.1 \quad \tilde{Y}_{12} = 2.3, \quad \tilde{I}_v = 0.08,$
$\tilde{Y}_2 = 50, \quad \tilde{H}_2 = 0.1, \quad \tilde{Y}_{2n} = 1.2e^{-7}, \quad \tilde{K}_{2n} = 0.035, \quad \tilde{F}_4 = 0.01, \quad \tilde{\alpha}_m = 1, \quad \tilde{\alpha}_c = 1, \quad \tilde{\alpha}_b = 1, \quad \tilde{\alpha}_f = 1,$
$\tilde{D}_f = 0.02, \quad \tilde{C}_{kf} = 0.4, \quad \tilde{K}_{kf} = 0.1, \quad \tilde{A}_{f0} = 0.1, \quad \tilde{K}_f = 0.1, \quad \tilde{A}_{fn} = 0.3, \quad \tilde{K}_{fn} = 0.09, \quad \tilde{B}_v = 0.08,$
$\tilde{B}_{ec} = 1.5, \quad \tilde{A}_{5n1} = 10, \quad \tilde{H}_{5n1} = 0.005, \quad \tilde{A}_{5n2} = 10, \quad \tilde{H}_{5n2} = 0.11, \quad \tilde{A}_{6n} = 10, \quad \tilde{H}_{6n} = 0.02, \quad \tilde{A}_{7n} = 10,$
$\tilde{H}_{7n} = 0.005, \quad \tilde{A}_{8n} = 10, \quad \tilde{H}_{8n} = 0.0225, \quad \tilde{Y}_3 = 2000, \quad \tilde{H}_3 = 0.1, \quad \tilde{A}_{c0} = 0.101, \quad \tilde{K}_c = 0.1,$
$\tilde{A}_{cn} = 3.5e^{-7}, \quad \tilde{K}_{cn} = 0.035, \quad \tilde{A}_{b0} = 0.202, \quad \tilde{K}_b = 0.1, \quad \tilde{A}_{bn} = 1, \quad \tilde{K}_{bn} = 0.08, \quad \tilde{\kappa}_f = 1, \quad \tilde{\kappa}_c = 1,$
$\tilde{\kappa}_b = 1, \quad \tilde{d}_b = 0.01, \quad \tilde{d}_f = 10, \quad \tilde{P}_{fs} = 0.2, \quad \tilde{Q}_f = 1.5, \quad \tilde{P}_{cs} = 0.2, \quad \tilde{Q}_c = 1.5, \quad \tilde{P}_{bs} = 2.0, \quad \tilde{D}_{gbc} = 0.005,$
$\tilde{G}_{gc} = 50, \quad \tilde{H}_{gc} = 1, \quad \tilde{K}_{gc} = 0.1, \quad \tilde{d}_{gbc} = 100, \quad \tilde{G}_{gb} = 1000, \quad \tilde{H}_{gb} = 1, \quad \tilde{D}_{gv} = 0.5, \quad \tilde{G}_{gvb} = 5e^{-5},$
$\tilde{H}_{gv} = 0.03, \quad \tilde{G}_{gvc} = 1000, \quad \tilde{d}_{gvc} = 1000, \quad \tilde{d}_{gv} = 25, \quad \tilde{Q}_{hyp,m} = 1, \quad \tilde{K}_{hyp,m} = 0.02, \quad \tilde{Q}_{hyp,\mathrm{c}} = 1,$
$\tilde{K}_{hyp,\mathrm{c}} = 0.015, \quad \tilde{Q}_{hyp,\mathrm{b}} = 1 \quad \tilde{K}_{hyp,\mathrm{b}} = 0.04, \quad \tilde{Q}_{hyp,\mathrm{f}} = 1, \quad \tilde{K}_{hyp,\mathrm{f}} = 0.045, \quad \tilde{D}_n = 0.014, \quad \tilde{G}_n = 2.2,$
$\tilde{H}_n = 0.11, \quad \tilde{Q}_{n,m} = 2, \quad \tilde{K}_{n,m} = 0.02, \quad \tilde{Q}_{n,\mathrm{c}} = 0.04, \quad \tilde{K}_{n,\mathrm{c}} = 0.015, \quad \tilde{Q}_{n,\mathrm{b}} = 2.2, \quad \tilde{K}_{n,\mathrm{b}} = 0.04,$
$\tilde{Q}_{n,\mathrm{f}} = 2.5, \quad \tilde{K}_{n,\mathrm{f}} = 0.045.$

Table S.1. Parameter values of EC state variables.

Parameter	Definition	Setting
M_{tot}	number of membrane agents per EC	1500
V_{max}	maximal amount of VEGFR-2 receptors	115000
V_{min}	minimal amount of VEGFR-2 receptors	2500
N	amount of Notch receptors (constant)	25000
D_{max}	maximal amount of Dll4	25000
A_{max}	maximal amount of actin	5000
V_{sink}	proportion of VEGF left by VEGFR-1	0.275
σ	VEGFR-2 expression change due to Notch1	47.40
δ	Dll4 expression change due to VEGFR-2	6.32
V'^*	threshold of active VEGFR-2 for actin production and migration of tip	200
A^*	threshold of actin for tip cell selection	3500
V^*	threshold of VEGFR-2 for tip cell selection	$V_{max}/2$
ΔA	constant increment of actin	50
D_1	delay between active and effective active VEGFR-2 levels	3.δt
D_2	delay between active and effective active Notch levels	3.δt
D_3	inactive time before retraction of filopodia	3.δt
ee	maximal time step of inner loop	1.2 h
row	maximal time step of outer loop	8.57 h

1.4 Boundary and initial conditions

The system of equations must be complemented by suitable **initial and boundary conditions** to ensure the existence, uniqueness and non-negativity of a solution. At the start of the simulation, the entire callus area was filled with a loose fibrous tissue matrix ($\tilde{m}$ = 0.1), osteochondrogenic growth factors ($\tilde{g}_{bc} = 1$), mesenchymal stem cells ($\tilde{c}_m = 0.02$) and fibroblasts ($\tilde{c}_f = 0.01$). Due to the rupturing of the blood vessels during the fracture, the oxygen tension in the fracture callus will gradually decrease. The following initial gradient of oxygen tension is considered: $\tilde{n}_{init} = 0.037 + 0.0825\tilde{x}$ with $\tilde{x}$ representing the non-dimensionalised coordinate on the horizontal axis. All other variables were assumed to be zero initially. The mathematical model was closed by prescribing suitable boundary conditions. No-flux boundary conditions were applied for all variables carrying diffusion or taxis terms in their equations, except for the situations described below, where Dirichlet boundary conditions (i.e. concentration assigned at the boundary mimicking presence of that variable outside of the simulation domain) were prescribed for certain components on specific parts of the boundary and for a specified period of time. Mesenchymal stem cells and fibroblasts were released into the callus tissue at the beginning of the healing process from three possible sources: the periosteum, the surrounding soft tissues and the marrow space at the site of the damaged cortical tissue [31]. All sources were adopted here ($\tilde{c}_{m\_bc}$ = 0.02 & $\tilde{c}_{f\_bc}$ = 0.02 during first 3 days). The tip cells are initialized at specific starting positions, reflecting the intact vasculature in the cortical bone and marrow cavity [31]. The osteochondrogenic growth factor was assumed to originate from the fractured bone ends and the cortex away from the fracture site. ($\tilde{g}_{bc}$ = 20 during respectively 5 and 10 days) [32,33].

Table S.2. Initial values of the endothelial cell state variables.

Variable	Definition	Setting
V	level of VEGFR-2	V_{max}
N	level of Notch1	N
D	level of Dll4	0
V'	level of active VEGFR-2	0
N'	level of active Notch1	0
V''	level of effective active VEGFR-2	0
N''	level of effective active Notch1	0
A	level of actin	5000

1.5 Implementation

The 10 continuous variables are non-negative. This qualitative property of the solution must be inherited by its numerically computed approximation because, amongst others, erroneous negative values for the concentrations might render otherwise stable reaction terms unstable. Besides ensuring non-negativity, the algorithm employed for the numerical solution of the model must respect conservation of mass. The finite volume technique was employed for its inherent mass conservation properties. The Method of Lines (MOL) was applied to separate the spatial and temporal discretization. The axi-symmetric structure of the problem was employed to reduce the model to an equivalent problem in 2D space, leading subsequently to an efficient spatial discretization. The spatial domain was covered with an equidistant computational grid. After convergence tests the grid size was fixed at 25 µm in both directions. On this grid, the diffusion and reaction terms in the system of equations (1-10) were discretized using respectively the standard second order central difference approximation and pointwise evaluation, which were found to be sufficient in terms of accuracy and to ensure non-negativity of the solution of the resulting ordinary differential equation (ODE) system [34]. Contrarily, the discretization of the taxis terms in this system of equations required the application of

upwinding techniques with nonlinear limiter functions (van Leer limiter) to guarantee accurate, non-negative solutions of the MOL-ODE system. The order of the spatial approximation is two in general. For the time integration of the resulting stiff MOL-ODE system the code ROWMAP [35] was used. The methods built-in automatic step size control ensures the error caused in each time step (local error) to remain below a user-prescribed tolerance while keeping the computational cost as low as possible.

Firstly the continuous variables are calculated. Then the inner loop is iterated which consists of four subroutines: (1) the current tip cells are evaluated by the tip cell selection criterion and, if necessary, they lose their tip cell phenotype; (2) the new position of every tip cell is calculated using a central difference scheme in space in combination with explicit Euler time integration; (3) the code checks whether sprouting occurs, meaning that other endothelial cells also satisfy the criterion for tip cell selection; (4) the intracellular levels of every endothelial cell are updated. Finally, the inner and outer loops are iterated until the end time of the simulation is reached. The outer loop has a maximal time step size of 8.57 hours (*row*). Since the tip cells do not move more than one grid cell (25 µm) in this time interval ($v_{tip}^{\max}$ = 35 µm/day [25]), this maximal time step size (*row*) implies that the 11 PDEs can be solved for a constant vasculature. The inner loop has a maximal step size of 1.2 hours (*ee*), similar to Peiffer et al. [24], and was chosen so that the movement of the tip cells within one grid cell could be accurately followed (*ee* << *row*). To reduce implementation difficulties, the time step of the inner loop (δt) is determined by calculating how many maximal inner loop time steps (*ee*) can fit in one outer loop time step (ΔT) and dividing the outer loop time step by this number. Consequently, the time step of the inner loop is not constant, which means that D_1, D_2 and D_3 vary slightly, but this

is a minor trade-off for the computational efficiency. Numerical convergence tests have shown that the average inner time step δt is equal to 155 s. Consequently, D_1, D_2, D_3 approximate the delays chosen by Bentley et al. [14]. Since the time step δt is approximately 10 times the time step of Bentley et al. [14], the parameter values of σ and δ have been altered to match the dynamics of the Dll4-Notch system. Numerical tests have shown that similar behavior is retrieved when both σ and δ are multiplied with 3.16.

For a discussion of the simplifications related to the methods described above, we refer the reader to Geris et al., Peiffer et al. and Carlier et al. [24,30,36,37].

2. Description of the NF1 model

To examine the effect of the *Nf1* mutation on bone fracture healing, the parameter values of the factors describing the aberrant cellular behaviour of *Nf1* haploinsufficient and *Nf1* bi-allelically inactivated cells are altered (Table S.3). A wide range of parameter values (Nf1 range) is investigated to account for the variable proportion of *Nf1* haploinsufficient and *Nf1* bi-allelically inactivated cells in the pseudarthrosis region. In particular, a larger deviation of the parameter values from the normal case represents a higher proportion of bi-allelically inactivated cells. We have investigated the influence of eight factors described in the literature as contributors to the poor fracture healing outcome in CPT. These factors include the increased invasion of fibrous lesion cells ($c_{f,BC}$) [38], increased fibroblastic proliferation (A_{f0}) [38], increased fibroblastic differentiation (F_4) [39], reduced osteogenic differentiation (Y_{11}) [38, 40], reduced endochondral ossification ($Y_{3,cb}$) [38], reduced cartilage formation (P_{mc}) [38], increased fibrous tissue formation (P_{mf}) [39], and increased angiogenic growth factor production (G_{gvc}) [38, 41].

The responses (i.e. model outcomes) that are studied in relation to the altered parameters are listed in Table S.4. In order to calculate the tissue fractions, the spatial images are first made binary using tissue-specific thresholds (0 means that the tissue is not present and 1 means that the tissue is present in a grid cell). Subsequently, an equal weight is assigned to the different tissues, i.e. if a grid cell contains three tissues, the area of that grid cell is divided by three in the final calculations of the tissue (area) fractions. Since a pseudarthrosis is defined by the tissues present in the fracture gap area, all the responses were calculated for this area. An additional response "CI" (complication index) was introduced to assess the degree of severity of CPT. More specifically, the CI (γ_7) combines two continuous responses (i.e. the amount of fibrous tissue γ_4 and fibroblasts γ_5, each varying between 0 and 1 for a low and high amount respectively) with a Boolean response γ_6 (i.e. bony union or non-union represented by 0 or 1 respectively):

$$\gamma_7 = \frac{\gamma_4 + \gamma_5 + \gamma_6}{3} \tag{S.35}$$

A typical parameter combination for which the value of the CI is small is one or which the degree of severity of CPT is small, or in other words, when the fracture healing proceeds fairly normally. Conversely, a typical parameter combination for which the value of the CI is large, is one for which the degree of severity of CPT is large, or in other words, when the fracture healing is severely impaired.

BMP treatment was modeled using one generic osteochondrogenic growth factor (g_{bc}), which represents the effect of multiple growth factors present in the fracture callus and released from the BMP sponge as a clinical treatment. The influence of this generic growth factor on

differentiation is either chondrogenic or osteogenic depending on the local oxygen tension. To model the gradual release of BMP from a BMP sponge, implanted directly after the occurrence of the fracture, a time-dependent, periosteal boundary condition was simulated (Eq. S.36):

$$g_{bc,BC} = g^{*}_{bc,BC} \cdot e^{-t/\tau} \qquad \text{(S.36)}$$

with τ the time constant of the exponential decay, chosen to be equal to 10 days [42,43]. $g^{*}_{bc,BC}$ is equal to the standard value of the osteochondrogenic boundary condition.

Table S.3. Parameter values of the factors describing the aberrant cellular behavior of the *Nf1* mutated cells.

Factor	Symbol	Normal case	Nf1 case	Nf1 range
Invasion time fibroblasts	$c_{f,BC}$	3	20	0-50
Fibroblastic proliferation	A_{f0}	0.1	2	0.1-10
Fibroblastic differentiation	F_4	0.01	0.2	0.01-1
Osteogenic differentiation	Y_{11}	20	1	0-20
Endochondral ossification	$Y_{3,cb}$	1000	50	0-1000
Cartilage formation	P_{mc}	0.2	0.02	0-0.2
Fibrous tissue formation	P_{mf}	0.2	2	0.2-10
Angiogenic growth factor production	G_{gvc}	10^3	5.10^4	10^3-10^5

Table S.4. Analysed responses and their range of possible values.

Response	Symbol	Type	Day 7	Day 21	Day 35	Day 49
Bone tissue fraction	γ_1	Continuous	0%-100%	0%-100%	0%-100%	0%-100%
Fibrous tissue fraction	γ_2	Continuous	0%-100%	0%-100%	0%-100%	0%-100%
Cartilage tissue fraction	γ_3	Continuous	0%-100%	0%-100%	0%-100%	0%-100%
Fibrous tissue	γ_4	Continuous	0-1	0-1	0-1	0-1
Fibroblasts	γ_5	Continuous	0-1	0-1	0-1	0-1
Non-union	γ_6	Boolean	0/1	0/1	0/1	0/1
CI	γ_7	Continuous	0-1	0-1	0-1	0-1

3. Additional information

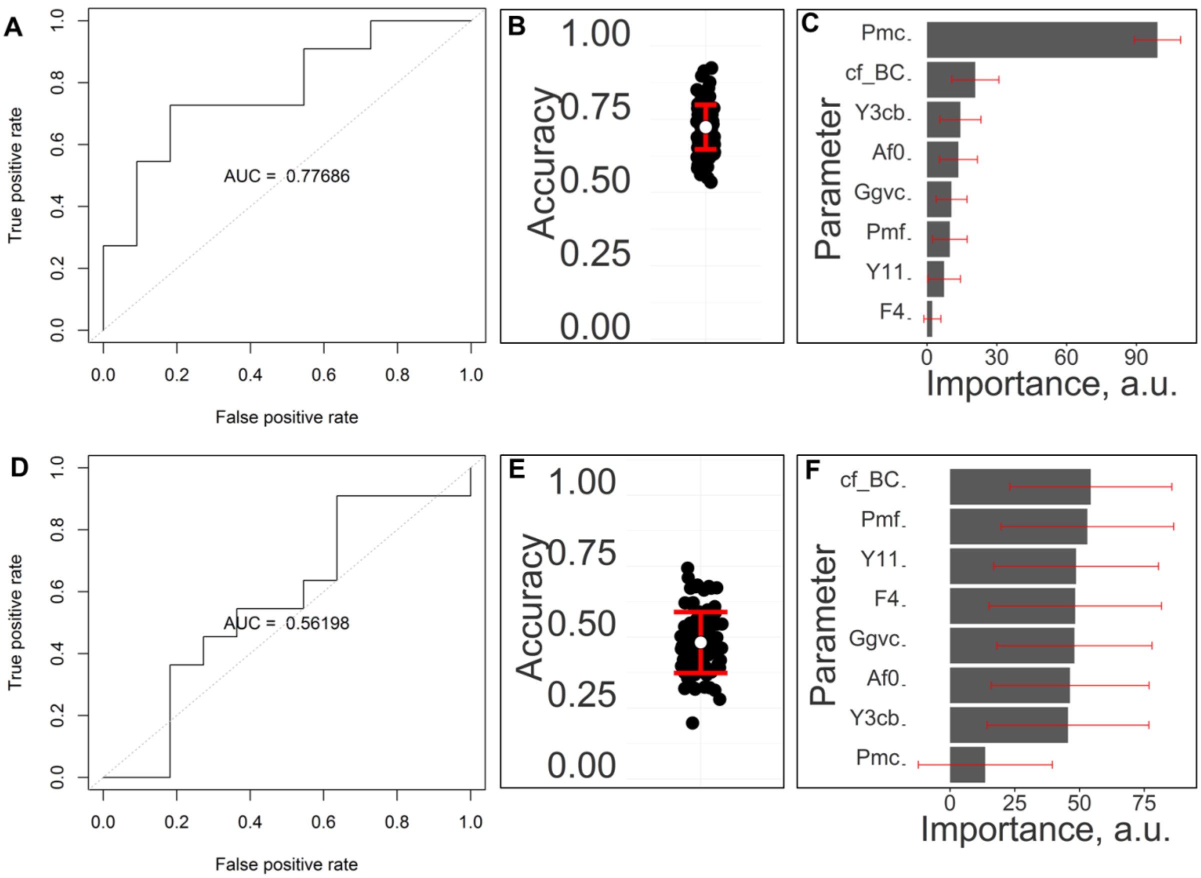

Figure S1: Overview of the results of the data-driven modeling. (A) Example of a ROC-curve regarding the classification between responders and non-responders, based on the eight NF1-related parameter values. (B) Accuracy of 100 predicted random forest models based on the eight NF1-related parameter values. (C) The importance of the eight NF1-related parameter values for predicting the patient class based on 100 random forest models. (D) Example of a ROC-curve based on scrambled data. (E) Accuracy of 100 predicted random forest models based on scrambled data. (F) The importance of the eight NF1-related parameter values for predicting the patient class based on 100 random forest models of scrambled data. a.u.: arbitrary units.

Table S5: Description of the NF1 factors and simulated outcomes listed in patients_data_2.csv

Factor	Symbol	Factor	Symbol
Invasion time fibroblasts	$c_{f,BC}$	Absolute amount of fibrous tissue at day 21	*abs_fibtis_d21*
Fibroblastic proliferation	A_{f0}	Fibrous tissue fraction at day 21	*fib_frac_d21*
Fibroblastic differentiation	F_4	Bone tissue fraction at day 21	*bone_frac_d21*
Osteogenic differentiation	Y_{11}	Cartilage tissue fraction at day 21	*cart_frac_d21*
Endochondral ossification	$Y_{3,cb}$	Fibroblast concentration at day 21	*fibroblasts_d21*
Cartilage formation	P_{mc}	Absolute amount of fibrous tissue at day 35	*abs_fibtis_d35*
Fibrous tissue formation	P_{mf}	Fibrous tissue fraction at day 35	*fib_frac_d35*
Angiogenic growth factor production	G_{gvc}	Bone tissue fraction at day 35	*bone_frac_d35*
Treatment (0 = no treatment, 1 = BMP treatment)		Cartilage tissue fraction at day 35	*cart_frac_d35*
Absolute amount of fibrous tissue at day 7	*abs_fibtis_d7*	Fibroblast concentration at day 35	*fibroblasts_d35*
Fibrous tissue fraction at day 7	*fib_frac_d7*	Absolute amount of fibrous tissue at day 49	*abs_fibtis_d49*
Bone tissue fraction at day 7	*bone_frac_d7*	Fibrous tissue fraction at day 49	*fib_frac_d49*
Cartilage tissue fraction at day 7	*cart_frac_d7*	Bone tissue fraction at day 49	*bone_frac_d49*
Fibroblast concentration at day 7	*fibroblasts_d7*	Cartilage tissue fraction at day 49	*cart_frac_d49*
Non-union at day 7	*NU_d7*	Fibroblast concentration at day 49	*fibroblasts_d49*
Non-union at day 21	*NU_d21*	Complication index at day 7	*CI_d7*
Non-union at day 35	*NU_d35*	Complication index at day 21	*CI_d21*
Non-union at day 49	*NU_d49*	Complication index at day 35	*CI_d35*
Complication index with treatment (is equal CI_d49 for treatment = 1)	*CI_withBMP*	Complication index at day 49	*CI_d49*
Complication index without treatment (is equal to CI_d49 for treatment = 0)	*CI_withoutBMP*		

In order to calculate the tissue fractions, the spatial results are first binarised using tissue-specific thresholds (0 means that the tissue is not present and 1 means that the tissue is present in a grid cell). Subsequently, an equal weight is assigned to the different tissues, i.e. if a grid cell contains three tissues, the area of that grid cell is divided by three in the final calculations of the

tissue (area) fractions. Since a pseudarthrosis is defined by the tissues present in the fracture gap area, all the responses are calculated for this area. An additional response “CI” (complication index) was introduced to assess the degree of severity of CPT. More specifically, the CI (γ_7) combines two continuous responses (i.e. the amount of fibrous tissue γ_4 and fibroblasts γ_5, each varying between 0 and 1 for a low and high amount respectively) with a Boolean response γ_6 (i.e. bony union or non-union represented by 0 or 1 respectively):

$$\gamma_7 = \frac{\gamma_4 + \gamma_5 + \gamma_6}{3}$$

A typical parameter combination for which the value of the CI is small is one for which the degree of severity of CPT is small, or in other words, the fracture healing proceeds fairly normally. Conversely, a typical parameter combination for which the value of the CI is large, is one for which the degree of severity of CPT is large, or in other words, the fracture healing is severely impaired. A non-union is defined as less than 10% of bone tissue fraction in the gap area.

4. Supplementary R-code

```
######SUPPLEMENTARY CODE##########

###Load all requered packages

if (!require("pacman")) install.packages("pacman")

pacman::p_load(ape, caret, ROCR,picante,plyr,doParallel, ggplot2, corrplot,randomForest,e1071)


##Load the data, correct path to the file should be specified

pat.data<-read.csv2(file="patients_data_2.csv",header=T, stringsAsFactors=F)


####################

##### FIGURE 2 #####

####################


hist(pat.data[1:200,c(38)],freq=TRUE, xlab="CI value", main ="", col=rgb(1,0,0,0.5))

hist(pat.data[1:200,c(39)],freq=TRUE, col=rgb(0,0,1,0.5), add=T)

box

legend("top", legend=c("CI without BMP", "CI with BMP", "overlap"), fill = c(rgb(1,0,0,0.5),
rgb(0,0,1,0.5),"purple"))


barplot(c(200-sum(pat.data[c(1:200), 33]), 200-sum(pat.data[c(201:400), 33])),

	ylab=("number of unions"), col=c(rgb(1,0,0,0.5), rgb(0,0,1,0.5)), names=c("without BMP","with
BMP"))

#legend("topleft",legend=c("without BMP","with BMP"),col=c(rgb(1,0,0,0.5), rgb(0,0,1,0.5)),

#	fill = c(rgb(0,0,1,0.5), rgb(1,0,0,0.5)))
```

```
# statistical testing on CI-index

CI_withoutBMP = pat.data[c(1:200), 38]

CI_withBMP = pat.data[c(1:200), 39]


t.test(CI_withoutBMP, CI_withBMP, paired=T)


boxplot(CI_withoutBMP, CI_withBMP, names=c("CI without BMP", "CI with BMP"), frame=F)

mtext("*", side=3, line=0, at=2, cex=1.2)


pat.data$Class<-"NoResp"

pat.data[pat.data$CI_withoutBMP>=0.5&pat.data$CI_withBMP<0.5,"Class"]<-"Responders"

pat.data[pat.data$CI_withoutBMP<0.5&pat.data$CI_withBMP<0.5,"Class"]<-"Asymp"

pat.data[pat.data$CI_withoutBMP<0.5&pat.data$CI_withBMP>0.5,"Class"]<-"AdversR"


table(pat.data$Class)

unique(pat.data$Class)


pat.data$Class<-as.factor(pat.data$Class)


tiff("scatter.tiff", height = 5, width = 7, units = 'in', res=600)


colors = c("red", "blue", "green", "black")

groups <- pat.data[,c(40)]

plot(pat.data[1:200,c(38)], pat.data[1:200,c(39)], col=colors[groups], xlab="CI without BMP", ylab="CI with BMP", pch=19, xlim=c(0,0.9), ylim=c(0,0.85))
```

```
#legend("topleft", legend = c("AdversR","noResp","Asymp","Responders"), col=c("red", "blue", "green", "black"), lwd=, lty=c(1,2))

#text(pat.data[1:200,c(38)], pat.data[1:200,c(39)], cex= 0.5, pos=4, col=colors[groups]) #labels=c(1:200)

dev.off()

par(mfrow = c(1,1))



tiff("dendrogram.tiff", height = 6, width = 6, units = 'in', res=600)


d <- dist(pat.data[1:200,c(38,39)], method = "euclidean") # distance matrix

fit <- hclust(d, method="ward.D2")

colors = c("red", "blue", "green", "black")

groups <- pat.data[1:200,c(40)]

tiplabels(text=pat.data[1:200,c(40)])

plot(as.phylo(fit), type = "fan", tip.color = colors[groups],  no.margin = TRUE,

    label.offset = 0, cex = 0.7)

#legend("topleft", legend = c("AdversR","noResp","Asymp","Responders"), col=c("red", "blue", "green", "black"), lwd=, lty=c(1,2))

dev.off()

par(mfrow = c(1,1))



####################

##### FIGURE 3 #####

####################

pat.data<-read.csv2("patients_data_2.csv",header=T, stringsAsFactors=F)
```

```
pat.data<-sapply(pat.data, as.numeric)

correlationMatrix <- cor(pat.data[c(1:200), -c(9,10,12,14,15,19,20,24,25,29,30,31,32,33,34,35,36,37)],
method="spearman");


tiff("scatter3.tiff", height = 4, width = 4, units = 'in', res=600)

corrplot(correlationMatrix, type="upper", order="original", tl.col="black", tl.srt=45, tl.cex=0.6,
number.cex=0.6)

dev.off()

par(mfrow = c(1,1))



####################

##### FIGURE 4 #####

####################

#pairwise t-test abs_fib_tis_d49_after value accross groups

pat.data<-read.csv2("patients_data_2.csv",header=T, stringsAsFactors=F)

pat.data$Class<-"NoResp"

pat.data[pat.data$CI_withoutBMP>=0.5&pat.data$CI_withBMP<0.5,"Class"]<-"Responders"

pat.data[pat.data$CI_withoutBMP<0.5&pat.data$CI_withBMP<0.5,"Class"]<-"Asymp"

pat.data[pat.data$CI_withoutBMP<0.5&pat.data$CI_withBMP>0.5,"Class"]<-"AdversR"


table(pat.data$Class)

unique(pat.data$Class)


pat.data$Class<-as.factor(pat.data$Class)
```

```
pat.data<-pat.data[201:400,]

pat.data1<-pat.data[pat.data$Class%in%c("AdversR"),]

pat.data2<-pat.data[pat.data$Class%in%c("NoResp"),]

pat.data3<-pat.data[pat.data$Class%in%c("Asymp"),]

pat.data4<-pat.data[pat.data$Class%in%c("Responders"),]

x1<-pat.data1[,c(25)]

x2<-pat.data2[,c(25)]

x3<-pat.data3[,c(25)]

x4<-pat.data4[,c(25)]

x <- data.frame(data=c(x1,x2,x3,x4),

        key=c(

         rep("x1", length(x1)),

         rep("x2", length(x2)),

         rep("x3", length(x3)),

         rep("x4", length(x4))) )


pairwise.t.test(x$data,

        x$key,

        pool.sd=FALSE, p.adjust.method="bonferroni")


# boxplot abs_fib_tis_day49_after_treatment

pat.data1<-pat.data[pat.data$Class%in%c("AdversR"),]

pat.data2<-pat.data[pat.data$Class%in%c("NoResp"),]

pat.data3<-pat.data[pat.data$Class%in%c("Asymp"),]

pat.data4<-pat.data[pat.data$Class%in%c("Responders"),]
```

```
boxplot(x1, x2, x3, x4, names=c("AdversR","noResp","Asymp","Responders"), frame=F, ylab="Fibrous tissue at day 49 - with treatment")



#non-union accross groups

x1<-1-pat.data1[,c(33)]

x2<-1-pat.data2[,c(33)]

x3<-1-pat.data3[,c(33)]

x4<-1-pat.data4[,c(33)]


x1<-pat.data1[,c(33)]/6

x2<-pat.data2[,c(33)]/47

x3<-pat.data3[,c(33)]/100

x4<-pat.data4[,c(33)]/47


barplot(height = cbind(sum(x1), sum(x2), sum(x3), sum(x4)),ylab=("fraction of non-unions per class"), names=c("AdversR","noResp", "Asymp", "Responders"))




###################################

##### Supplementary Figure S1 #####

###################################

# Train model, Responders versus non-responders

set.seed(107)

pat.data.select<-pat.data[1:200,]
```

```
pat.data2<-pat.data.select[pat.data.select$Class%in%c("NoResp","Responders"),]
pat.data2$Class<-"NoResp"
pat.data2[pat.data2$CI_withoutBMP>=0.5&pat.data2$CI_withBMP<0.5,"Class"]<-"Responders"
pat.data2$Class<-as.factor(pat.data2$Class)
inTrain <- createDataPartition(y = pat.data2$Class,p = .75, list = FALSE)
training <- pat.data2[ inTrain,]
testing <- pat.data2[-inTrain,]
nrow(training)
nrow(testing)

cvCtrl <- trainControl(method = "cv", number = 10, classProbs = TRUE)
rfTune <- train(training[,1:8], training[,40], method = "rf",
                tuneLength = 30,
                metric = "Accuracy",
                trControl = cvCtrl)

rfProbs <- predict(rfTune, testing[,-40])#, type = "prob")
confusionMatrix(rfProbs, testing$Class)
rfProbs <- predict(rfTune, testing, type = "prob")[,2]

pred <- prediction(rfProbs, testing$Class)
perf <- performance(pred,"tpr","fpr")
plot(perf,col="black",lty=3, lwd=3)
abline(a=0,b=1,col="grey",lwd=1,lty=3)

tiff("Figure_supp_A.tiff", height = 5, width = 5, units = 'in', res=600)
```

```
perf_AUC=performance(pred,"auc") #Calculate the AUC value
AUC=perf_AUC@y.values[[1]]
perf_ROC=performance(pred,"tpr","fpr") #plot the actual ROC curve

plot(perf_ROC, main="ROC plot")
text(0.5,0.5,paste("AUC = ",format(AUC, digits=5, scientific=FALSE)))
abline(a=0,b=1,col="grey",lwd=1,lty=3)

# test prediction random forests multiple times noRespH - responders
class_data<-pat.data2[,"Class"]
model_accuracy<-data.frame(Run=c(),Accuracy=c(),BalAccuracy=c(),ROC=c())
##create empty matrix for feature importance
NF1.predictors<-data.frame(t(pat.data2[1,]))
NF1.predictors$X1<-0
for(i in 1:100)
{

  inTrain <- createDataPartition(y = pat.data2$Class,p = .75, list = FALSE)
  training <- pat.data2[ inTrain,]
  testing <- pat.data2[-inTrain,]
  nrow(training)
  nrow(testing)

  cvCtrl <- trainControl(method = "cv", number = 10, classProbs = TRUE)
```

```
cl <- makeCluster(detectCores(), type='PSOCK') #specify number of cores

registerDoParallel(cl)


rfTune <- train(training[,1:8], training[,40], method = "rf",

          tuneLength = 30,

          metric = "Accuracy",

          allowParallel=TRUE,

          trControl = cvCtrl)


stopCluster(cl)

registerDoSEQ()


NF1.predictors.temp<-data.frame(varImp(rfTune)$importance)

NF1.predictors<-cbind(NF1.predictors,NF1.predictors.temp[,][match(rownames(NF1.predictors),
rownames(NF1.predictors.temp))])

colnames(NF1.predictors)[ncol(NF1.predictors)]<-paste("trial",i)


rfPred <- predict(rfTune, testing[,-40])#, type = "prob")

confusionMatrix(rfPred, testing$Class)


rfProbs <- predict(rfTune, testing, type = "prob")[,2]




pred <- prediction(rfProbs, testing$Class)

perf <- performance(pred,"tpr","fpr")
```

```
  accuracy<-confusionMatrix(rfPred, testing$Class)
  accuracy[[3]][1]
  confmat<-table(rfPred, testing$Class)

  auc<-performance(pred,"auc")
  auc <- unlist(slot(auc, "y.values"))

  balanced_accuracy<-((confmat[2,2]/sum(confmat[,2]))+confmat[1,1]/sum(confmat[,1]))/2
  balanced_accuracy
  auc
  results<-c(i,as.numeric(accuracy[[3]][1]),balanced_accuracy,auc)
  model_accuracy<-rbind(model_accuracy,results)
}

colnames(model_accuracy)<-c("Trial","Accuracy","Balanced accuracy","ROC")
model_accuracy

boxplot(model_accuracy[,-1])
accu_mean<-mean(model_accuracy[,2])
accu_sd<-sd(model_accuracy[,2])

ggplot(model_accuracy, aes(Accuracy,x="Accuracy")) + geom_jitter(width = 0.2, cex=5)+
  ylim(0,1.2)+theme_bw()+
  geom_errorbar(aes(ymin =accu_mean-accu_sd, ymax = accu_mean+accu_sd),
                colour = "red", width = 0.2, cex=2)+
```

```
  geom_point(aes(accu_mean,x="Accuracy"), size=5, shape=21, fill="white")+
  theme_minimal()+
  ylab("Accuracy")+theme(#text = element_text(size=18),
    axis.text.x = element_blank(),
    axis.text.y = element_text(colour="grey20",size=32,angle=0,hjust=1,vjust=0,face="plain"),
    axis.title.x = element_blank(),
    axis.title.y = element_text(colour="grey20",size=32,angle=90,hjust=.5,vjust=.5,face="plain"))

ggsave("Figure_SB.tiff", units="in", width=3, height=5, dpi=600)

##show combined feature importance
NF1.predictors.f<-NF1.predictors[-nrow(NF1.predictors),-1]
##calculate statistics


NF1.pred.stat<-transform(NF1.predictors.f, SD=apply(NF1.predictors.f,1, sd, na.rm = TRUE),
                         Mean=apply(NF1.predictors.f,1, mean, na.rm = TRUE))
NF1.pred.stat.s<-NF1.pred.stat[order(-NF1.pred.stat$Mean),]
NF1.pred.stat.s<-NF1.pred.stat.s[1:8,] #number of rows

NF1.pred.stat.s$Cond<-row.names(NF1.pred.stat.s)
NF1.pred.stat.s$Order<-c(nrow(NF1.pred.stat.s):1)
NF1.pred.stat.s$Order<-as.factor(NF1.pred.stat.s$Order)

ggplot(NF1.pred.stat.s, aes(y=Mean, x=Order)) +
  geom_bar(stat="identity", position=position_dodge()) +
```

```
  geom_errorbar(aes(ymin=Mean-SD, ymax=Mean+SD), width=.2, cex=0.4,
              position=position_dodge(.9),colour="red")+
  scale_x_discrete(breaks=c(1:nrow(NF1.pred.stat.s)),
                labels=rev(as.character(NF1.pred.stat.s$Cond)))+
  coord_flip() +  theme_classic() +  xlab("Parameter")+
  ylab("Importance, a.u.")+theme(#text = element_text(size=18),
    axis.text.x = element_text(colour="grey20",size=20,angle=0,hjust=.5,vjust=.5,face="plain"),
    axis.text.y = element_text(colour="grey20",size=20,angle=0,hjust=1,vjust=0,face="plain"),
    axis.title.x = element_text(colour="grey20",size=32,angle=0,hjust=.5,vjust=0,face="plain"),
    axis.title.y = element_text(colour="grey20",size=32,angle=90,hjust=.5,vjust=.5,face="plain"))

ggsave("Figure_SC.tiff", units="in", width=5, height=5, dpi=600)

# scrambled data
set.seed(107)
pat.data2[,c(1,2,3,4,5,6,7,8)]<-randomizeMatrix(pat.data2[,c(1,2,3,4,5,6,7,8)],null.model =
"frequency",iterations = 1000)

pat.data2$Class<-as.factor(pat.data2$Class)

inTrain <- createDataPartition(y = pat.data2$Class,p = .75, list = FALSE)
training <- pat.data2[ inTrain,]
testing <- pat.data2[-inTrain,]
nrow(training)
nrow(testing)
```

```
cvCtrl <- trainControl(method = "cv", number = 10, classProbs = TRUE)
rfTune <- train(training[,1:8], training[,40], method = "rf",
                tuneLength = 30,
                metric = "Accuracy",
                trControl = cvCtrl)

rfProbs <- predict(rfTune, testing[,-40])#, type = "prob")
confusionMatrix(rfProbs, testing$Class)

rfProbs <- predict(rfTune, testing, type = "prob")[,2]


pred <- prediction(rfProbs, testing$Class)
perf <- performance(pred,"tpr","fpr")
plot(perf,col="black",lty=3, lwd=3)
abline(a=0,b=1,col="grey",lwd=1,lty=3)

tiff("Figure_supp_D.tiff", height = 5, width = 5, units = 'in', res=600)

perf_AUC=performance(pred,"auc") #Calculate the AUC value
AUC=perf_AUC@y.values[[1]]
perf_ROC=performance(pred,"tpr","fpr") #plot the actual ROC curve
plot(perf_ROC, main="ROC plot")
text(0.5,0.5,paste("AUC = ",format(AUC, digits=5, scientific=FALSE)))
abline(a=0,b=1,col="grey",lwd=1,lty=3)
```

```
# test scrambled data multiple times

  model_accuracy<-data.frame(Run=c(),Accuracy=c(),BalAccuracy=c(),ROC=c())

  NF1.predictors<-data.frame(t(pat.data2[1,]))

  NF1.predictors$X1<-0

  for(i in 1:100)

  {

    pat.data2[,c(1,2,3,4,5,6,7,8)]<-randomizeMatrix(pat.data2[,c(1,2,3,4,5,6,7,8)],null.model =
"frequency",iterations = 1000)

    pat.data2$Class<-as.factor(pat.data2$Class)


    inTrain <- createDataPartition(y = pat.data2$Class,p = .75, list = FALSE)

    training <- pat.data2[ inTrain,]

    testing <- pat.data2[-inTrain,]

    nrow(training)

    nrow(testing)


    cvCtrl <- trainControl(method = "cv", number = 10, classProbs = TRUE)




    cl <- makeCluster(detectCores(), type='PSOCK') #specify number of cores

    registerDoParallel(cl)


    rfTune <- train(training[,1:8], training[,40], method = "rf",

                   tuneLength = 30,

                   metric = "Accuracy",

                   allowParallel=TRUE,
```

```
                  trControl = cvCtrl)

  stopCluster(cl)
  registerDoSEQ()

  NF1.predictors.temp<-data.frame(varImp(rfTune)$importance)
  NF1.predictors<-cbind(NF1.predictors,NF1.predictors.temp[,][match(rownames(NF1.predictors), rownames(NF1.predictors.temp))])
  colnames(NF1.predictors)[ncol(NF1.predictors)]<-paste("trial",i)

  rfPred <- predict(rfTune, testing[,-11])#, type = "prob")
  confusionMatrix(rfPred, testing$Class)

  rfProbs <- predict(rfTune, testing, type = "prob")[,2]



  pred <- prediction(rfProbs, testing$Class)
  perf <- performance(pred,"tpr","fpr")

  accuracy<-confusionMatrix(rfPred, testing$Class)
  accuracy[[3]][1]
  confmat<-table(rfPred, testing$Class)

  auc<-performance(pred,"auc")
  auc <- unlist(slot(auc, "y.values"))
```

```
  balanced_accuracy<-((confmat[2,2]/sum(confmat[,2]))+confmat[1,1]/sum(confmat[,1]))/2
  balanced_accuracy
  auc
  results<-c(i,as.numeric(accuracy[[3]][1]),balanced_accuracy,auc)
  model_accuracy<-rbind(model_accuracy,results)
}

colnames(model_accuracy)<-c("Trial","Accuracy","Balanced accuracy","ROC")
model_accuracy
boxplot(model_accuracy[,-1])
accu_mean<-mean(model_accuracy[,2])
accu_sd<-sd(model_accuracy[,2])



ggplot(model_accuracy, aes(Accuracy,x="Accuracy")) + geom_jitter(width = 0.2, cex=5)+
  ylim(0,1.2)+theme_bw()+
  geom_errorbar(aes(ymin =accu_mean-accu_sd, ymax = accu_mean+accu_sd),
                colour = "red", width = 0.2, cex=2)+
  geom_point(aes(accu_mean,x="Accuracy"), size=5, shape=21, fill="white")+
  theme_minimal()+
  ylab("Accuracy")+theme(#text = element_text(size=18),
    axis.text.x = element_blank(),
    axis.text.y = element_text(colour="grey20",size=32,angle=0,hjust=1,vjust=0,face="plain"),
    axis.title.x = element_blank(),
    axis.title.y = element_text(colour="grey20",size=32,angle=90,hjust=.5,vjust=.5,face="plain"))
```

```
ggsave("Figure_SE.tiff", units="in", width=5, height=5, dpi=600)


##show combined feature importance
NF1.predictors.f<-NF1.predictors[-nrow(NF1.predictors),-1]
##calculate statistics


NF1.pred.stat<-transform(NF1.predictors.f, SD=apply(NF1.predictors.f,1, sd, na.rm = TRUE),
                         Mean=apply(NF1.predictors.f,1, mean, na.rm = TRUE))
NF1.pred.stat.s<-NF1.pred.stat[order(-NF1.pred.stat$Mean),]
NF1.pred.stat.s<-NF1.pred.stat.s[1:8,] #number of rows


NF1.pred.stat.s$Cond<-row.names(NF1.pred.stat.s)
NF1.pred.stat.s$Order<-c(nrow(NF1.pred.stat.s):1)
NF1.pred.stat.s$Order<-as.factor(NF1.pred.stat.s$Order)


ggplot(NF1.pred.stat.s, aes(y=Mean, x=Order)) +
  geom_bar(stat="identity", position=position_dodge()) +
  geom_errorbar(aes(ymin=Mean-SD, ymax=Mean+SD), width=.2, cex=0.4,
                position=position_dodge(.9),colour="red")+
  scale_x_discrete(breaks=c(1:nrow(NF1.pred.stat.s)),
                   labels=rev(as.character(NF1.pred.stat.s$Cond)))+
  coord_flip() +  theme_classic() +  xlab("Parameter")+
  ylab("Importance, a.u.")+theme(#text = element_text(size=18),
    axis.text.x = element_text(colour="grey20",size=20,angle=0,hjust=.5,vjust=.5,face="plain"),
    axis.text.y = element_text(colour="grey20",size=20,angle=0,hjust=1,vjust=0,face="plain"),
    axis.title.x = element_text(colour="grey20",size=32,angle=0,hjust=.5,vjust=0,face="plain"),
```

```
    axis.title.y = element_text(colour="grey20",size=32,angle=90,hjust=.5,vjust=.5,face="plain"))
  ggsave("Figure_SF.tiff", units="in", width=5, height=5, dpi=600)




####################
##### TABLE 1  #####
####################

# responders (47 patients) – non-responders (47 patients)
######################################################################
set.seed(107)
pat.data<-read.csv2("patients_data_2.csv",header=T, stringsAsFactors=F)
pat.data$Class<-"NoResp"
pat.data[pat.data$CI_withoutBMP>=0.5&pat.data$CI_withBMP<0.5,"Class"]<-"Responders"
pat.data[pat.data$CI_withoutBMP<0.5&pat.data$CI_withBMP<0.5,"Class"]<-"Asymp"
pat.data[pat.data$CI_withoutBMP<0.5&pat.data$CI_withBMP>0.5,"Class"]<-"AdversR"

table(pat.data$Class)
unique(pat.data$Class)

pat.data$Class<-as.factor(pat.data$Class)

pat.data.select<-pat.data[1:200,]
```

```
pat.data2<-pat.data.select[pat.data.select$Class%in%c("NoResp","Responders"),]
pat.data2$Class<-"NoResp"
pat.data2[pat.data2$CI_withoutBMP>=0.5&pat.data2$CI_withBMP<0.5,"Class"]<-"Responders"
pat.data2$Class<-as.factor(pat.data2$Class)
class_data<-pat.data2[,"Class"]
model_accuracy<-data.frame(Run=c(),Accuracy=c(),BalAccuracy=c(),ROC=c())
NF1.predictors<-data.frame(t(pat.data2[1,]))
NF1.predictors$X1<-0
for(i in 1:100)
{

  inTrain <- createDataPartition(y = pat.data2$Class,p = .75, list = FALSE)
  training <- pat.data2[ inTrain,]
  testing <- pat.data2[-inTrain,]
  nrow(training)
  nrow(testing)

  cvCtrl <- trainControl(method = "cv", number = 10, classProbs = TRUE)



  cl <- makeCluster(detectCores(), type='PSOCK') #specify number of cores
  registerDoParallel(cl)

  rfTune <- train(training[,1:8], training[,40], method = "rf",
                  tuneLength = 30,
                  metric = "Accuracy",
```

```
                   allowParallel=TRUE,
                   trControl = cvCtrl)

  stopCluster(cl)
  registerDoSEQ()

  NF1.predictors.temp<-data.frame(varImp(rfTune)$importance)
  NF1.predictors<-cbind(NF1.predictors,NF1.predictors.temp[,][match(rownames(NF1.predictors),
rownames(NF1.predictors.temp))])
  colnames(NF1.predictors)[ncol(NF1.predictors)]<-paste("trial",i)

  rfPred <- predict(rfTune, testing[,-11])#, type = "prob")
  confusionMatrix(rfPred, testing$Class)

  rfProbs <- predict(rfTune, testing, type = "prob")[,2]



  pred <- prediction(rfProbs, testing$Class)
  perf <- performance(pred,"tpr","fpr")

  accuracy<-confusionMatrix(rfPred, testing$Class)
  accuracy[[3]][1]
  confmat<-table(rfPred, testing$Class)

  auc<-performance(pred,"auc")
  auc <- unlist(slot(auc, "y.values"))
```

```
  balanced_accuracy<-((confmat[2,2]/sum(confmat[,2]))+confmat[1,1]/sum(confmat[,1]))/2
  balanced_accuracy
  auc
  results<-c(i,as.numeric(accuracy[[3]][1]),balanced_accuracy,auc)
  model_accuracy<-rbind(model_accuracy,results)
}

colnames(model_accuracy)<-c("Trial","Accuracy","Balanced accuracy","ROC")
model_accuracy
boxplot(model_accuracy[,-1])
accu_mean<-mean(model_accuracy[,2])
accu_sd<-sd(model_accuracy[,2])


ggplot(model_accuracy, aes(Accuracy,x="Accuracy")) + geom_jitter(width = 0.2, cex=5)+
  ylim(0.4,1)+theme_bw()+
  geom_errorbar(aes(ymin =accu_mean-accu_sd, ymax = accu_mean+accu_sd),
                colour = "red", width = 0.2, cex=2)+
  geom_point(aes(accu_mean,x="Accuracy"), size=5, shape=21, fill="white")+
  theme_minimal()+
  ylab("Accuracy")+theme(#text = element_text(size=18),
    axis.text.x = element_blank(),
    axis.text.y = element_text(colour="grey20",size=32,angle=0,hjust=1,vjust=0,face="plain"),
    axis.title.x = element_blank(),
    axis.title.y = element_text(colour="grey20",size=32,angle=90,hjust=.5,vjust=.5,face="plain"))
```

```
##show combined feature importance
NF1.predictors.f<-NF1.predictors[-nrow(NF1.predictors),-1]
##calculate statistics

NF1.pred.stat<-transform(NF1.predictors.f, SD=apply(NF1.predictors.f,1, sd, na.rm = TRUE),
                         Mean=apply(NF1.predictors.f,1, mean, na.rm = TRUE))
NF1.pred.stat.s<-NF1.pred.stat[order(-NF1.pred.stat$Mean),]
#NF1.pred.stat.s<-NF1.pred.stat.s[(nrow(NF1.pred.stat.s)-20):nrow(NF1.pred.stat.s),]
NF1.pred.stat.s<-NF1.pred.stat.s[1:8,] #number of rows

NF1.pred.stat.s$Cond<-row.names(NF1.pred.stat.s)
NF1.pred.stat.s$Order<-c(nrow(NF1.pred.stat.s):1)
NF1.pred.stat.s$Order<-as.factor(NF1.pred.stat.s$Order)
#levels(NF1.pred.stat.s$Cond)<-as.character(NF1.pred.stat.s$Cond)

#levels(NF1.pred.stat.s$Cond)<-row.names(NF1.pred.stat.s)

ggplot(NF1.pred.stat.s, aes(y=Mean, x=Order)) +
  geom_bar(stat="identity", position=position_dodge()) +
  geom_errorbar(aes(ymin=Mean-SD, ymax=Mean+SD), width=.2, cex=0.4,
                position=position_dodge(.9),colour="red")+
  scale_x_discrete(breaks=c(1:nrow(NF1.pred.stat.s)),
                   labels=rev(as.character(NF1.pred.stat.s$Cond)))+
  coord_flip() +  theme_classic() +  xlab("Parameter")+
  ylab("Importance, a.u.")+theme(#text = element_text(size=18),
    axis.text.x = element_text(colour="grey20",size=20,angle=0,hjust=.5,vjust=.5,face="plain"),
```

```
    axis.text.y = element_text(colour="grey20",size=20,angle=0,hjust=1,vjust=0,face="plain"),
    axis.title.x = element_text(colour="grey20",size=32,angle=0,hjust=.5,vjust=0,face="plain"),
    axis.title.y = element_text(colour="grey20",size=32,angle=90,hjust=.5,vjust=.5,face="plain"))


# responders (47 patients) – asymptomatic (100 patients)
######################################################################
set.seed(107)
pat.data<-read.csv2("patients_data_2.csv",header=T, stringsAsFactors=F)
pat.data$Class<-"NoResp"
pat.data[pat.data$CI_withoutBMP>=0.5&pat.data$CI_withBMP<0.5,"Class"]<-"Responders"
pat.data[pat.data$CI_withoutBMP<0.5&pat.data$CI_withBMP<0.5,"Class"]<-"Asymp"
pat.data[pat.data$CI_withoutBMP<0.5&pat.data$CI_withBMP>0.5,"Class"]<-"AdversR"

table(pat.data$Class)
unique(pat.data$Class)

pat.data$Class<-as.factor(pat.data$Class)

pat.data.select<-pat.data[1:200,]

pat.data2<-pat.data.select[pat.data.select$Class%in%c("Asymp","Responders"),]
pat.data2$Class<-"Responders"
pat.data2[pat.data2$CI_withoutBMP<=0.5&pat.data2$CI_withBMP<=0.5,"Class"]<-"Asymp"
pat.data2$Class<-as.factor(pat.data2$Class)
class_data<-pat.data2[,"Class"]
```

```
model_accuracy<-data.frame(Run=c(),Accuracy=c(),BalAccuracy=c(),ROC=c())

NF1.predictors<-data.frame(t(pat.data2[1,]))

NF1.predictors$X1<-0

for(i in 1:100)

{


 inTrain <- createDataPartition(y = pat.data2$Class,p = .75, list = FALSE)

 training <- pat.data2[ inTrain,]

 testing <- pat.data2[-inTrain,]

 nrow(training)

 nrow(testing)


 cvCtrl <- trainControl(method = "cv", number = 10, classProbs = TRUE)




 cl <- makeCluster(detectCores(), type='PSOCK') #specify number of cores

 registerDoParallel(cl)


 rfTune <- train(training[,1:8], training[,40], method = "rf",

                  tuneLength = 30,

                  metric = "Accuracy",

                  allowParallel=TRUE,

                  trControl = cvCtrl)


 stopCluster(cl)

 registerDoSEQ()
```

```
  NF1.predictors.temp<-data.frame(varImp(rfTune)$importance)
  NF1.predictors<-cbind(NF1.predictors,NF1.predictors.temp[,][match(rownames(NF1.predictors),
rownames(NF1.predictors.temp))])
  colnames(NF1.predictors)[ncol(NF1.predictors)]<-paste("trial",i)

  rfPred <- predict(rfTune, testing[,-11])#, type = "prob")
  confusionMatrix(rfPred, testing$Class)

  rfProbs <- predict(rfTune, testing, type = "prob")[,2]


  pred <- prediction(rfProbs, testing$Class)
  perf <- performance(pred,"tpr","fpr")

  accuracy<-confusionMatrix(rfPred, testing$Class)
  accuracy[[3]][1]
  confmat<-table(rfPred, testing$Class)

  auc<-performance(pred,"auc")
  auc <- unlist(slot(auc, "y.values"))

  balanced_accuracy<-((confmat[2,2]/sum(confmat[,2]))+confmat[1,1]/sum(confmat[,1]))/2
  balanced_accuracy
  auc
  results<-c(i,as.numeric(accuracy[[3]][1]),balanced_accuracy,auc)
```

```
  model_accuracy<-rbind(model_accuracy,results)
}

colnames(model_accuracy)<-c("Trial","Accuracy","Balanced accuracy","ROC")
model_accuracy
boxplot(model_accuracy[,-1])
accu_mean<-mean(model_accuracy[,2])
accu_sd<-sd(model_accuracy[,2])



ggplot(model_accuracy, aes(Accuracy,x="Accuracy")) + geom_jitter(width = 0.2, cex=5)+
  ylim(0.4,1)+theme_bw()+
  geom_errorbar(aes(ymin =accu_mean-accu_sd, ymax = accu_mean+accu_sd),
               colour = "red", width = 0.2, cex=2)+
  geom_point(aes(accu_mean,x="Accuracy"), size=5, shape=21, fill="white")+
  theme_minimal()+
  ylab("Accuracy")+theme(#text = element_text(size=18),
    axis.text.x = element_blank(),
    axis.text.y = element_text(colour="grey20",size=32,angle=0,hjust=1,vjust=0,face="plain"),
    axis.title.x = element_blank(),
    axis.title.y = element_text(colour="grey20",size=32,angle=90,hjust=.5,vjust=.5,face="plain"))
##show combined feature importance
NF1.predictors.f<-NF1.predictors[-nrow(NF1.predictors),-1]
##calculate statistics

NF1.pred.stat<-transform(NF1.predictors.f, SD=apply(NF1.predictors.f,1, sd, na.rm = TRUE),
```

```
                        Mean=apply(NF1.predictors.f,1, mean, na.rm = TRUE))
NF1.pred.stat.s<-NF1.pred.stat[order(-NF1.pred.stat$Mean),]
#NF1.pred.stat.s<-NF1.pred.stat.s[(nrow(NF1.pred.stat.s)-20):nrow(NF1.pred.stat.s),]
NF1.pred.stat.s<-NF1.pred.stat.s[1:8,] #number of rows

NF1.pred.stat.s$Cond<-row.names(NF1.pred.stat.s)
NF1.pred.stat.s$Order<-c(nrow(NF1.pred.stat.s):1)
NF1.pred.stat.s$Order<-as.factor(NF1.pred.stat.s$Order)
#levels(NF1.pred.stat.s$Cond)<-as.character(NF1.pred.stat.s$Cond)

#levels(NF1.pred.stat.s$Cond)<-row.names(NF1.pred.stat.s)

ggplot(NF1.pred.stat.s, aes(y=Mean, x=Order)) +
  geom_bar(stat="identity", position=position_dodge()) +
  geom_errorbar(aes(ymin=Mean-SD, ymax=Mean+SD), width=.2, cex=0.4,
              position=position_dodge(.9),colour="red")+
  scale_x_discrete(breaks=c(1:nrow(NF1.pred.stat.s)),
                labels=rev(as.character(NF1.pred.stat.s$Cond)))+
  coord_flip() +  theme_classic() +  xlab("Parameter")+
  ylab("Importance, a.u.")+theme(#text = element_text(size=18),
    axis.text.x = element_text(colour="grey20",size=20,angle=0,hjust=.5,vjust=.5,face="plain"),
    axis.text.y = element_text(colour="grey20",size=20,angle=0,hjust=1,vjust=0,face="plain"),
    axis.title.x = element_text(colour="grey20",size=32,angle=0,hjust=.5,vjust=0,face="plain"),
    axis.title.y = element_text(colour="grey20",size=32,angle=90,hjust=.5,vjust=.5,face="plain"))
```

```
# Asymptomatic (100 patients) – non-responders (47 patients)

#####################################################################

set.seed(107)

pat.data<-read.csv2("patients_data_2.csv",header=T, stringsAsFactors=F)

pat.data$Class<-"NoResp"

pat.data[pat.data$CI_withoutBMP>=0.5&pat.data$CI_withBMP<0.5,"Class"]<-"Responders"

pat.data[pat.data$CI_withoutBMP<0.5&pat.data$CI_withBMP<0.5,"Class"]<-"Asymp"

pat.data[pat.data$CI_withoutBMP<0.5&pat.data$CI_withBMP>0.5,"Class"]<-"AdversR"


table(pat.data$Class)

unique(pat.data$Class)


pat.data$Class<-as.factor(pat.data$Class)


pat.data.select<-pat.data[1:200,]


pat.data2<-pat.data.select[pat.data.select$Class%in%c("Asymp","NoResp"),]

pat.data2$Class<-"noResp"

pat.data2[pat.data2$CI_withoutBMP<=0.5&pat.data2$CI_withBMP<=0.5,"Class"]<-"Asymp"

pat.data2$Class<-as.factor(pat.data2$Class)

class_data<-pat.data2[,"Class"]

model_accuracy<-data.frame(Run=c(),Accuracy=c(),BalAccuracy=c(),ROC=c())

NF1.predictors<-data.frame(t(pat.data2[1,]))

NF1.predictors$X1<-0

for(i in 1:100)

{
```

```
inTrain <- createDataPartition(y = pat.data2$Class,p = .75, list = FALSE)

training <- pat.data2[ inTrain,]

testing <- pat.data2[-inTrain,]

nrow(training)

nrow(testing)


cvCtrl <- trainControl(method = "cv", number = 10, classProbs = TRUE)




cl <- makeCluster(detectCores(), type='PSOCK') #specify number of cores

registerDoParallel(cl)


rfTune <- train(training[,1:8], training[,40], method = "rf",

                tuneLength = 30,

                metric = "Accuracy",

                allowParallel=TRUE,

                trControl = cvCtrl)


stopCluster(cl)

registerDoSEQ()


NF1.predictors.temp<-data.frame(varImp(rfTune)$importance)

NF1.predictors<-cbind(NF1.predictors,NF1.predictors.temp[,][match(rownames(NF1.predictors),
rownames(NF1.predictors.temp))])

colnames(NF1.predictors)[ncol(NF1.predictors)]<-paste("trial",i)
```

```
  rfPred <- predict(rfTune, testing[,-11])#, type = "prob")
  confusionMatrix(rfPred, testing$Class)

  rfProbs <- predict(rfTune, testing, type = "prob")[,2]


  pred <- prediction(rfProbs, testing$Class)
  perf <- performance(pred,"tpr","fpr")

  accuracy<-confusionMatrix(rfPred, testing$Class)
  accuracy[[3]][1]
  confmat<-table(rfPred, testing$Class)

  auc<-performance(pred,"auc")
  auc <- unlist(slot(auc, "y.values"))

  balanced_accuracy<-((confmat[2,2]/sum(confmat[,2]))+confmat[1,1]/sum(confmat[,1]))/2
  balanced_accuracy
  auc
  results<-c(i,as.numeric(accuracy[[3]][1]),balanced_accuracy,auc)
  model_accuracy<-rbind(model_accuracy,results)
}

colnames(model_accuracy)<-c("Trial","Accuracy","Balanced accuracy","ROC")
model_accuracy
```

```
boxplot(model_accuracy[,-1])

accu_mean<-mean(model_accuracy[,2])

accu_sd<-sd(model_accuracy[,2])



ggplot(model_accuracy, aes(Accuracy,x="Accuracy")) + geom_jitter(width = 0.2, cex=5)+
  ylim(0.4,1)+theme_bw()+
  geom_errorbar(aes(ymin =accu_mean-accu_sd, ymax = accu_mean+accu_sd),
              colour = "red", width = 0.2, cex=2)+
  geom_point(aes(accu_mean,x="Accuracy"), size=5, shape=21, fill="white")+
  theme_minimal()+
  ylab("Accuracy")+theme(#text = element_text(size=18),
    axis.text.x = element_blank(),
    axis.text.y = element_text(colour="grey20",size=32,angle=0,hjust=1,vjust=0,face="plain"),
    axis.title.x = element_blank(),
    axis.title.y = element_text(colour="grey20",size=32,angle=90,hjust=.5,vjust=.5,face="plain"))
##show combined feature importance
NF1.predictors.f<-NF1.predictors[-nrow(NF1.predictors),-1]
##calculate statistics

NF1.pred.stat<-transform(NF1.predictors.f, SD=apply(NF1.predictors.f,1, sd, na.rm = TRUE),
                        Mean=apply(NF1.predictors.f,1, mean, na.rm = TRUE))
NF1.pred.stat.s<-NF1.pred.stat[order(-NF1.pred.stat$Mean),]
#NF1.pred.stat.s<-NF1.pred.stat.s[(nrow(NF1.pred.stat.s)-20):nrow(NF1.pred.stat.s),]
NF1.pred.stat.s<-NF1.pred.stat.s[1:8,] #number of rows
```

```
NF1.pred.stat.s$Cond<-row.names(NF1.pred.stat.s)

NF1.pred.stat.s$Order<-c(nrow(NF1.pred.stat.s):1)

NF1.pred.stat.s$Order<-as.factor(NF1.pred.stat.s$Order)

#levels(NF1.pred.stat.s$Cond)<-as.character(NF1.pred.stat.s$Cond)


#levels(NF1.pred.stat.s$Cond)<-row.names(NF1.pred.stat.s)


ggplot(NF1.pred.stat.s, aes(y=Mean, x=Order)) +
  geom_bar(stat="identity", position=position_dodge()) +
  geom_errorbar(aes(ymin=Mean-SD, ymax=Mean+SD), width=.2, cex=0.4,
              position=position_dodge(.9),colour="red")+
  scale_x_discrete(breaks=c(1:nrow(NF1.pred.stat.s)),
                labels=rev(as.character(NF1.pred.stat.s$Cond)))+
  coord_flip() +  theme_classic() +  xlab("Parameter")+
  ylab("Importance, a.u.")+theme(#text = element_text(size=18),
    axis.text.x = element_text(colour="grey20",size=20,angle=0,hjust=.5,vjust=.5,face="plain"),
    axis.text.y = element_text(colour="grey20",size=20,angle=0,hjust=1,vjust=0,face="plain"),
    axis.title.x = element_text(colour="grey20",size=32,angle=0,hjust=.5,vjust=0,face="plain"),
    axis.title.y = element_text(colour="grey20",size=32,angle=90,hjust=.5,vjust=.5,face="plain"))



# responders (47 patients) – adverse responders (6 patients)
############################################################
set.seed(107)
pat.data<-read.csv2("patients_data_2.csv",header=T, stringsAsFactors=F)
pat.data$Class<-"NoResp"
```

```
pat.data[pat.data$CI_withoutBMP>=0.5&pat.data$CI_withBMP<0.5,"Class"]<-"Responders"
pat.data[pat.data$CI_withoutBMP<0.5&pat.data$CI_withBMP<0.5,"Class"]<-"Asymp"
pat.data[pat.data$CI_withoutBMP<0.5&pat.data$CI_withBMP>0.5,"Class"]<-"AdversR"

table(pat.data$Class)
unique(pat.data$Class)

pat.data$Class<-as.factor(pat.data$Class)

pat.data.select<-pat.data[1:200,]

pat.data2<-pat.data.select[pat.data.select$Class%in%c("Responders","AdversR"),]
pat.data2$Class<-"Responders"
pat.data2[pat.data2$CI_withoutBMP<=0.5&pat.data2$CI_withBMP>0.5,"Class"]<-"AdversR"
pat.data2$Class<-as.factor(pat.data2$Class)
class_data<-pat.data2[,"Class"]
model_accuracy<-data.frame(Run=c(),Accuracy=c(),BalAccuracy=c(),ROC=c())
NF1.predictors<-data.frame(t(pat.data2[1,]))
NF1.predictors$X1<-0
for(i in 1:100)
{

  inTrain <- createDataPartition(y = pat.data2$Class,p = .75, list = FALSE)
  training <- pat.data2[ inTrain,]
  testing <- pat.data2[-inTrain,]
  nrow(training)
```

```
nrow(testing)

cvCtrl <- trainControl(method = "cv", number = 10, classProbs = TRUE)


cl <- makeCluster(detectCores(), type='PSOCK') #specify number of cores
registerDoParallel(cl)

rfTune <- train(training[,1:8], training[,40], method = "rf",
          tuneLength = 30,
          metric = "Accuracy",
          allowParallel=TRUE,
          trControl = cvCtrl)

stopCluster(cl)
registerDoSEQ()

NF1.predictors.temp<-data.frame(varImp(rfTune)$importance)
NF1.predictors<-cbind(NF1.predictors,NF1.predictors.temp[,][match(rownames(NF1.predictors),
rownames(NF1.predictors.temp))])
colnames(NF1.predictors)[ncol(NF1.predictors)]<-paste("trial",i)

rfPred <- predict(rfTune, testing[,-11])#, type = "prob")
confusionMatrix(rfPred, testing$Class)

rfProbs <- predict(rfTune, testing, type = "prob")[,2]
```

```
  pred <- prediction(rfProbs, testing$Class)
  perf <- performance(pred,"tpr","fpr")

  accuracy<-confusionMatrix(rfPred, testing$Class)
  accuracy[[3]][1]
  confmat<-table(rfPred, testing$Class)

  auc<-performance(pred,"auc")
  auc <- unlist(slot(auc, "y.values"))

  balanced_accuracy<-((confmat[2,2]/sum(confmat[,2]))+confmat[1,1]/sum(confmat[,1]))/2
  balanced_accuracy
  auc
  results<-c(i,as.numeric(accuracy[[3]][1]),balanced_accuracy,auc)
  model_accuracy<-rbind(model_accuracy,results)
}

colnames(model_accuracy)<-c("Trial","Accuracy","Balanced accuracy","ROC")
model_accuracy
boxplot(model_accuracy[,-1])
accu_mean<-mean(model_accuracy[,2])
accu_sd<-sd(model_accuracy[,2])
```

```
ggplot(model_accuracy, aes(Accuracy,x="Accuracy")) + geom_jitter(width = 0.2, cex=5)+
  ylim(0.4,1)+theme_bw()+
  geom_errorbar(aes(ymin =accu_mean-accu_sd, ymax = accu_mean+accu_sd),
              colour = "red", width = 0.2, cex=2)+
  geom_point(aes(accu_mean,x="Accuracy"), size=5, shape=21, fill="white")+
  theme_minimal()+
  ylab("Accuracy")+theme(#text = element_text(size=18),
    axis.text.x = element_blank(),
    axis.text.y = element_text(colour="grey20",size=32,angle=0,hjust=1,vjust=0,face="plain"),
    axis.title.x = element_blank(),
    axis.title.y = element_text(colour="grey20",size=32,angle=90,hjust=.5,vjust=.5,face="plain"))
##show combined feature importance
NF1.predictors.f<-NF1.predictors[-nrow(NF1.predictors),-1]
##calculate statistics

NF1.pred.stat<-transform(NF1.predictors.f, SD=apply(NF1.predictors.f,1, sd, na.rm = TRUE),
                         Mean=apply(NF1.predictors.f,1, mean, na.rm = TRUE))
NF1.pred.stat.s<-NF1.pred.stat[order(-NF1.pred.stat$Mean),]
#NF1.pred.stat.s<-NF1.pred.stat.s[(nrow(NF1.pred.stat.s)-20):nrow(NF1.pred.stat.s),]
NF1.pred.stat.s<-NF1.pred.stat.s[1:8,] #number of rows

NF1.pred.stat.s$Cond<-row.names(NF1.pred.stat.s)
NF1.pred.stat.s$Order<-c(nrow(NF1.pred.stat.s):1)
NF1.pred.stat.s$Order<-as.factor(NF1.pred.stat.s$Order)
#levels(NF1.pred.stat.s$Cond)<-as.character(NF1.pred.stat.s$Cond)
```

```
#levels(NF1.pred.stat.s$Cond)<-row.names(NF1.pred.stat.s)

ggplot(NF1.pred.stat.s, aes(y=Mean, x=Order)) +
  geom_bar(stat="identity", position=position_dodge()) +
  geom_errorbar(aes(ymin=Mean-SD, ymax=Mean+SD), width=.2, cex=0.4,
              position=position_dodge(.9),colour="red")+
  scale_x_discrete(breaks=c(1:nrow(NF1.pred.stat.s)),
                labels=rev(as.character(NF1.pred.stat.s$Cond)))+
  coord_flip() +  theme_classic() +  xlab("Parameter")+
  ylab("Importance, a.u.")+theme(#text = element_text(size=18),
    axis.text.x = element_text(colour="grey20",size=20,angle=0,hjust=.5,vjust=.5,face="plain"),
    axis.text.y = element_text(colour="grey20",size=20,angle=0,hjust=1,vjust=0,face="plain"),
    axis.title.x = element_text(colour="grey20",size=32,angle=0,hjust=.5,vjust=0,face="plain"),
    axis.title.y = element_text(colour="grey20",size=32,angle=90,hjust=.5,vjust=.5,face="plain"))


# non-responders (47 patients) –  adverse responders (6 patients)
##############################################################################
set.seed(107)
pat.data<-read.csv2("patients_data_2.csv",header=T, stringsAsFactors=F)
pat.data$Class<-"NoResp"
pat.data[pat.data$CI_withoutBMP>=0.5&pat.data$CI_withBMP<0.5,"Class"]<-"Responders"
pat.data[pat.data$CI_withoutBMP<0.5&pat.data$CI_withBMP<0.5,"Class"]<-"Asymp"
pat.data[pat.data$CI_withoutBMP<0.5&pat.data$CI_withBMP>0.5,"Class"]<-"AdversR"

table(pat.data$Class)
```

```
unique(pat.data$Class)


pat.data$Class<-as.factor(pat.data$Class)


pat.data.select<-pat.data[1:200,]


pat.data2<-pat.data.select[pat.data.select$Class%in%c("NoResp","AdversR"),]
pat.data2$Class<-"NoResp"
pat.data2[pat.data2$CI_withoutBMP<=0.5&pat.data2$CI_withBMP>0.5,"Class"]<-"AdversR"
pat.data2$Class<-as.factor(pat.data2$Class)
class_data<-pat.data2[,"Class"]
model_accuracy<-data.frame(Run=c(),Accuracy=c(),BalAccuracy=c(),ROC=c())
NF1.predictors<-data.frame(t(pat.data2[1,]))
NF1.predictors$X1<-0
for(i in 1:100)
{

  inTrain <- createDataPartition(y = pat.data2$Class,p = .75, list = FALSE)
  training <- pat.data2[ inTrain,]
  testing <- pat.data2[-inTrain,]
  nrow(training)
  nrow(testing)

  cvCtrl <- trainControl(method = "cv", number = 10, classProbs = TRUE)
```

```
cl <- makeCluster(detectCores(), type='PSOCK') #specify number of cores

registerDoParallel(cl)


rfTune <- train(training[,1:8], training[,40], method = "rf",

          tuneLength = 30,

          metric = "Accuracy",

          allowParallel=TRUE,

          trControl = cvCtrl)


stopCluster(cl)

registerDoSEQ()


NF1.predictors.temp<-data.frame(varImp(rfTune)$importance)

NF1.predictors<-cbind(NF1.predictors,NF1.predictors.temp[,][match(rownames(NF1.predictors),
rownames(NF1.predictors.temp))])

colnames(NF1.predictors)[ncol(NF1.predictors)]<-paste("trial",i)


rfPred <- predict(rfTune, testing[,-11])#, type = "prob")

confusionMatrix(rfPred, testing$Class)


rfProbs <- predict(rfTune, testing, type = "prob")[,2]


pred <- prediction(rfProbs, testing$Class)

perf <- performance(pred,"tpr","fpr")
```

```
  accuracy<-confusionMatrix(rfPred, testing$Class)
  accuracy[[3]][1]
  confmat<-table(rfPred, testing$Class)

  auc<-performance(pred,"auc")
  auc <- unlist(slot(auc, "y.values"))

  balanced_accuracy<-((confmat[2,2]/sum(confmat[,2]))+confmat[1,1]/sum(confmat[,1]))/2
  balanced_accuracy
  auc
  results<-c(i,as.numeric(accuracy[[3]][1]),balanced_accuracy,auc)
  model_accuracy<-rbind(model_accuracy,results)
}

colnames(model_accuracy)<-c("Trial","Accuracy","Balanced accuracy","ROC")
model_accuracy
boxplot(model_accuracy[,-1])
accu_mean<-mean(model_accuracy[,2])
accu_sd<-sd(model_accuracy[,2])


ggplot(model_accuracy, aes(Accuracy,x="Accuracy")) + geom_jitter(width = 0.2, cex=5)+
  ylim(0.4,1)+theme_bw()+
  geom_errorbar(aes(ymin =accu_mean-accu_sd, ymax = accu_mean+accu_sd),
                colour = "red", width = 0.2, cex=2)+
  geom_point(aes(accu_mean,x="Accuracy"), size=5, shape=21, fill="white")+
```

```
  theme_minimal()+
  ylab("Accuracy")+theme(#text = element_text(size=18),
    axis.text.x = element_blank(),
    axis.text.y = element_text(colour="grey20",size=32,angle=0,hjust=1,vjust=0,face="plain"),
    axis.title.x = element_blank(),
    axis.title.y = element_text(colour="grey20",size=32,angle=90,hjust=.5,vjust=.5,face="plain"))
##show combined feature importance
NF1.predictors.f<-NF1.predictors[-nrow(NF1.predictors),-1]
##calculate statistics

NF1.pred.stat<-transform(NF1.predictors.f, SD=apply(NF1.predictors.f,1, sd, na.rm = TRUE),
                         Mean=apply(NF1.predictors.f,1, mean, na.rm = TRUE))
NF1.pred.stat.s<-NF1.pred.stat[order(-NF1.pred.stat$Mean),]
#NF1.pred.stat.s<-NF1.pred.stat.s[(nrow(NF1.pred.stat.s)-20):nrow(NF1.pred.stat.s),]
NF1.pred.stat.s<-NF1.pred.stat.s[1:8,] #number of rows

NF1.pred.stat.s$Cond<-row.names(NF1.pred.stat.s)
NF1.pred.stat.s$Order<-c(nrow(NF1.pred.stat.s):1)
NF1.pred.stat.s$Order<-as.factor(NF1.pred.stat.s$Order)
#levels(NF1.pred.stat.s$Cond)<-as.character(NF1.pred.stat.s$Cond)

#levels(NF1.pred.stat.s$Cond)<-row.names(NF1.pred.stat.s)

ggplot(NF1.pred.stat.s, aes(y=Mean, x=Order)) +
  geom_bar(stat="identity", position=position_dodge()) +
  geom_errorbar(aes(ymin=Mean-SD, ymax=Mean+SD), width=.2, cex=0.4,
```

```
              position=position_dodge(.9),colour="red")+
  scale_x_discrete(breaks=c(1:nrow(NF1.pred.stat.s)),
               labels=rev(as.character(NF1.pred.stat.s$Cond)))+
  coord_flip() +  theme_classic() +  xlab("Parameter")+
  ylab("Importance, a.u.")+theme(#text = element_text(size=18),
    axis.text.x = element_text(colour="grey20",size=20,angle=0,hjust=.5,vjust=.5,face="plain"),
    axis.text.y = element_text(colour="grey20",size=20,angle=0,hjust=1,vjust=0,face="plain"),
    axis.title.x = element_text(colour="grey20",size=32,angle=0,hjust=.5,vjust=0,face="plain"),
    axis.title.y = element_text(colour="grey20",size=32,angle=90,hjust=.5,vjust=.5,face="plain"))


# asymptomatic (100 patients) –  adverse responders (6 patients)
################################################################################
set.seed(107)
pat.data<-read.csv2("patients_data_2.csv",header=T, stringsAsFactors=F)
pat.data$Class<-"NoResp"
pat.data[pat.data$CI_withoutBMP>=0.5&pat.data$CI_withBMP<0.5,"Class"]<-"Responders"
pat.data[pat.data$CI_withoutBMP<0.5&pat.data$CI_withBMP<0.5,"Class"]<-"Asymp"
pat.data[pat.data$CI_withoutBMP<0.5&pat.data$CI_withBMP>0.5,"Class"]<-"AdversR"

table(pat.data$Class)
unique(pat.data$Class)

pat.data$Class<-as.factor(pat.data$Class)

pat.data.select<-pat.data[1:200,]
```

```
pat.data2<-pat.data.select[pat.data.select$Class%in%c("Asymp","AdversR"),]
pat.data2$Class<-"Asymp"
pat.data2[pat.data2$CI_withoutBMP<=0.5&pat.data2$CI_withBMP>0.5,"Class"]<-"AdversR"
pat.data2$Class<-as.factor(pat.data2$Class)
class_data<-pat.data2[,"Class"]
model_accuracy<-data.frame(Run=c(),Accuracy=c(),BalAccuracy=c(),ROC=c())
NF1.predictors<-data.frame(t(pat.data2[1,]))
NF1.predictors$X1<-0
for(i in 1:100)
{

 inTrain <- createDataPartition(y = pat.data2$Class,p = .75, list = FALSE)
 training <- pat.data2[ inTrain,]
 testing <- pat.data2[-inTrain,]
 nrow(training)
 nrow(testing)

 cvCtrl <- trainControl(method = "cv", number = 10, classProbs = TRUE)



 cl <- makeCluster(detectCores(), type='PSOCK') #specify number of cores
 registerDoParallel(cl)

 rfTune <- train(training[,1:8], training[,40], method = "rf",
          tuneLength = 30,
```

```
                 metric = "Accuracy",
                 allowParallel=TRUE,
                 trControl = cvCtrl)

  stopCluster(cl)
  registerDoSEQ()

  NF1.predictors.temp<-data.frame(varImp(rfTune)$importance)
  NF1.predictors<-cbind(NF1.predictors,NF1.predictors.temp[,][match(rownames(NF1.predictors),
rownames(NF1.predictors.temp))])
  colnames(NF1.predictors)[ncol(NF1.predictors)]<-paste("trial",i)

  rfPred <- predict(rfTune, testing[,-11])#, type = "prob")
  confusionMatrix(rfPred, testing$Class)

  rfProbs <- predict(rfTune, testing, type = "prob")[,2]


  pred <- prediction(rfProbs, testing$Class)
  perf <- performance(pred,"tpr","fpr")

  accuracy<-confusionMatrix(rfPred, testing$Class)
  accuracy[[3]][1]
  confmat<-table(rfPred, testing$Class)

  auc<-performance(pred,"auc")
```

```
  auc <- unlist(slot(auc, "y.values"))

  balanced_accuracy<-((confmat[2,2]/sum(confmat[,2]))+confmat[1,1]/sum(confmat[,1]))/2
  balanced_accuracy
  auc
  results<-c(i,as.numeric(accuracy[[3]][1]),balanced_accuracy,auc)
  model_accuracy<-rbind(model_accuracy,results)
}

colnames(model_accuracy)<-c("Trial","Accuracy","Balanced accuracy","ROC")
model_accuracy
boxplot(model_accuracy[,-1])
accu_mean<-mean(model_accuracy[,2])
accu_sd<-sd(model_accuracy[,2])


ggplot(model_accuracy, aes(Accuracy,x="Accuracy")) + geom_jitter(width = 0.2, cex=5)+
  ylim(0.4,1)+theme_bw()+
  geom_errorbar(aes(ymin =accu_mean-accu_sd, ymax = accu_mean+accu_sd),
              colour = "red", width = 0.2, cex=2)+
  geom_point(aes(accu_mean,x="Accuracy"), size=5, shape=21, fill="white")+
  theme_minimal()+
  ylab("Accuracy")+theme(#text = element_text(size=18),
    axis.text.x = element_blank(),
    axis.text.y = element_text(colour="grey20",size=32,angle=0,hjust=1,vjust=0,face="plain"),
    axis.title.x = element_blank(),
```

```
    axis.title.y = element_text(colour="grey20",size=32,angle=90,hjust=.5,vjust=.5,face="plain"))
##show combined feature importance
NF1.predictors.f<-NF1.predictors[-nrow(NF1.predictors),-1]
##calculate statistics

NF1.pred.stat<-transform(NF1.predictors.f, SD=apply(NF1.predictors.f,1, sd, na.rm = TRUE),
                         Mean=apply(NF1.predictors.f,1, mean, na.rm = TRUE))
NF1.pred.stat.s<-NF1.pred.stat[order(-NF1.pred.stat$Mean),]
#NF1.pred.stat.s<-NF1.pred.stat.s[(nrow(NF1.pred.stat.s)-20):nrow(NF1.pred.stat.s),]
NF1.pred.stat.s<-NF1.pred.stat.s[1:8,] #number of rows

NF1.pred.stat.s$Cond<-row.names(NF1.pred.stat.s)
NF1.pred.stat.s$Order<-c(nrow(NF1.pred.stat.s):1)
NF1.pred.stat.s$Order<-as.factor(NF1.pred.stat.s$Order)
#levels(NF1.pred.stat.s$Cond)<-as.character(NF1.pred.stat.s$Cond)

#levels(NF1.pred.stat.s$Cond)<-row.names(NF1.pred.stat.s)

ggplot(NF1.pred.stat.s, aes(y=Mean, x=Order)) +
  geom_bar(stat="identity", position=position_dodge()) +
  geom_errorbar(aes(ymin=Mean-SD, ymax=Mean+SD), width=.2, cex=0.4,
                position=position_dodge(.9),colour="red")+
  scale_x_discrete(breaks=c(1:nrow(NF1.pred.stat.s)),
                   labels=rev(as.character(NF1.pred.stat.s$Cond)))+
  coord_flip() +  theme_classic() +  xlab("Parameter")+
  ylab("Importance, a.u.")+theme(#text = element_text(size=18),
```

```
axis.text.x = element_text(colour="grey20",size=20,angle=0,hjust=.5,vjust=.5,face="plain"),

axis.text.y = element_text(colour="grey20",size=20,angle=0,hjust=1,vjust=0,face="plain"),

axis.title.x = element_text(colour="grey20",size=32,angle=0,hjust=.5,vjust=0,face="plain"),

axis.title.y = element_text(colour="grey20",size=32,angle=90,hjust=.5,vjust=.5,face="plain"))
```

5. References

1. Pountos I, Giannoudis PV (2005) Biology of mesenchymal stem cells. Injury-International Journal of the Care of the Injured 36: 8-12.

2. Fiedler J, Leucht F, Waltenberger J, Dehio C, Brenner RE (2005) VEGF-A and PlGF-1 stimulate chemotactic migration of human mesenchymal progenitor cells. Biochemical and Biophysical Research Communications 334: 561-568.

3. Olsen L, Sherratt JA, Maini PK, Arnold F (1997) A mathematical model for the capillary endothelial cell-extracellular matrix interactions in wound-healing angiogenesis. Ima Journal of Mathematics Applied in Medicine and Biology 14: 261-281.

4. Sherratt JA (1994) Chemotaxis and Chemokinesis in Eukaryotic Cells - the Keller-Segel Equations As An Approximation to A Detailed Model. Bulletin of Mathematical Biology 56: 129-146.

5. Weinberg CB, Bell E (1985) Regulation of Proliferation of Bovine Aortic Endothelial-Cells, Smooth-Muscle Cells, and Adventitial Fibroblasts in Collagen Lattices. Journal of Cellular Physiology 122: 410-414.

6. Gerstenfeld LC, Cullinane DM, Barnes GL, Graves DT, Einhorn TA (2003) Fracture healing as a post-natal developmental process: molecular, spatial, and temporal aspects of its regulation. J Cell Biochem 88: 873-884. 10.1002/jcb.10435 [doi].

7. Cho TJ, Gerstenfeld LC, Einhorn TA (2002) Differential temporal expression of members of the transforming growth factor beta superfamily during murine fracture healing. Journal of Bone and Mineral Research 17: 513-520.

8. Hirao M, Tamai N, Tsumaki N, Yoshikawa H, Myoui A (2006) Oxygen tension regulates chondrocyte differentiation and function during endochondral ossification. Journal of Biological Chemistry 281: 31079-31092.

9. Bailon-Plaza A, van der Meulen MC (2001) A mathematical framework to study the effects of growth factor influences on fracture healing. J Theor Biol 212: 191-209. 10.1006/jtbi.2001.2372 [doi];S0022-5193(01)92372-0 [pii].

10. Rossi F, MacLean HE, Yuan W, Francis RO, Semenova E, Lin CS, Kronenberg HM, Cobrinik D (2002) p107 and p130 coordinately regulate proliferation, Cbfa1 expression, and hypertrophic differentiation during endochondral bone development. Developmental Biology 247: 271-285.

11. Einhorn TA (1998) The cell and molecular biology of fracture healing. Clinical Orthopaedics and Related Research S7-S21.

12. Demol J, Lambrechts D, Geris L, Schrooten J, Van OH (2011) Towards a quantitative understanding of oxygen tension and cell density evolution in fibrin hydrogels. Biomaterials 32: 107-118. S0142-9612(10)01131-2 [pii];10.1016/j.biomaterials.2010.08.093 [doi].

13. Henrotin Y, Kurz B, Aigner T (2005) Oxygen and reactive oxygen species in cartilage degradation: friends or foes? Osteoarthritis and Cartilage 13: 643-654.

14. Bentley K, Gerhardt H, Bates PA (2008) Agent-based simulation of notch-mediated tip cell selection in angiogenic sprout initialisation. J Theor Biol 250: 25-36. S0022-5193(07)00443-2 [pii];10.1016/j.jtbi.2007.09.015 [doi].

15. Tillmann U, Bereiterhahn J (1986) Relation of Actin Fibrils to Energy-Metabolism of Endothelial-Cells. Cell and Tissue Research 243: 579-585.

16. Waingeh VF, Gustafson CD, Kozliak EI, Lowe SL, Knull HR, Thomasson KA (2006) Glycolytic enzyme interactions with yeast and skeletal muscle F-actin. Biophysical Journal 90: 1371-1384.

17. De Smet F, Segura I, De Bock K, Hohensinner PJ, Carmeliet P (2009) Mechanisms of Vessel Branching Filopodia on Endothelial Tip Cells Lead the Way. Arteriosclerosis Thrombosis and Vascular Biology 29: 639-649.

18. Potente M, Gerhardt H, Carmeliet P (2011) Basic and Therapeutic Aspects of Angiogenesis. Cell 146: 873-887.

19. Zhang JH, Fukuhara S, Sako K, Takenouchi T, Kitani H, Kume T, Koh GY, Mochizuki N (2011) Angiopoietin-1/Tie2 Signal Augments Basal Notch Signal Controlling Vascular Quiescence by Inducing Delta-Like 4 Expression through AKT-mediated Activation of beta-Catenin. Journal of Biological Chemistry 286: 8055-8066.

20. Yan MH, Callahan CA, Beyer JC, Allamneni KP, Zhang G, Ridgway JB, Niessen K, Plowman GD (2010) Chronic DLL4 blockade induces vascular neoplasms. Nature 463: E6-E7.

21. Giudicelli F, Lewis J (2004) The vertebrate segmentation clock. Current Opinion in Genetics & Development 14: 407-414.

22. Roca C, Adams RH (2007) Regulation of vascular morphogenesis by Notch signaling. Genes & Development 21: 2511-2524.

23. Hellstrom M, Phng LK, Hofmann JJ, Wallgard E, Coultas L, Lindblom P, Alva J, Nilsson AK, Karlsson L, Gaiano N, Yoon K, Rossant J, Iruela-Arispe ML, Kalen M, Gerhardt H, Betsholtz C (2007) Dll4 signalling through Notch1 regulates formation of tip cells during angiogenesis. Nature 445: 776-780.

24. Peiffer V, Gerisch A, Vandepitte D, Van OH, Geris L (2011) A hybrid bioregulatory model of angiogenesis during bone fracture healing. Biomech Model Mechanobiol 10: 383-395. 10.1007/s10237-010-0241-7 [doi].

25. Sun SY, Wheeler MF, Obeyesekere M, Patrick CW (2005) A deterministic model of growth factor-induced angiogenesis. Bulletin of Mathematical Biology 67: 313-337.

26. Phng LK, Gerhardt H (2009) Angiogenesis: A Team Effort Coordinated by Notch. Developmental Cell 16: 196-208.

27. Harrison LJ, Cunningham JL, Stromberg L, Goodship AE (2003) Controlled induction of a pseudarthrosis: A study using a rodent model. Journal of Orthopaedic Trauma 17: 11-21.

28. Nogami H, Oohira A (1984) Postnatal New Bone-Formation. Clinical Orthopaedics and Related Research 106-113.

29. Joyce ME, Terek RM, Jingushi S, Bolander ME (1990) Role of Transforming Growth-Factor-Beta in Fracture Repair. Annals of the New York Academy of Sciences 593: 107-123.

30. Geris L, Gerisch A, Sloten JV, Weiner R, Oosterwyck HV (2008) Angiogenesis in bone fracture healing: a bioregulatory model. J Theor Biol 251: 137-158. S0022-5193(07)00567-X [pii];10.1016/j.jtbi.2007.11.008 [doi].

31. Gerstenfeld LC, Cullinane DM, Barnes GL, Graves DT, Einhorn TA (2003) Fracture healing as a post-natal developmental process: Molecular, spatial, and temporal aspects of its regulation. Journal of Cellular Biochemistry 88: 873-884.

32. Barnes GL, Kostenuik PJ, Gerstenfeld LC, Einhorn TA (1999) Growth factor regulation of fracture repair. Journal of Bone and Mineral Research 14: 1805-1815.

33. Dimitriou R, Tsiridis E, Giannoudis PV (2005) Current concepts of molecular aspects of bone healing. Injury-International Journal of the Care of the Injured 36: 1392-1404.

34. Gerisch A, Chaplain MAJ (2006) Robust numerical methods for taxis-diffusion-reaction systems: Applications to biomedical problems. Mathematical and Computer Modelling 43: 49-75.

35. Weiner R, Schmitt BA, Podhaisky H (1997) ROWMAP - a ROW-code with Krylov techniques for large stiff ODEs. Applied Numerical Mathematics 25: 303-319.

36. Carlier A, Geris L, Bentley K, Carmeliet G, Carmeliet P, Van OH (2012) MOSAIC: a multiscale model of osteogenesis and sprouting angiogenesis with lateral inhibition of endothelial cells. PLoS Comput Biol 8: e1002724. 10.1371/journal.pcbi.1002724 [doi];PCOMPBIOL-D-12-00618 [pii].

37. Carlier A, Geris L, van Gastel N, Carmeliet G, Van Oosterwyck H (2014) Oxgen as a critical determinant of bone fracture healing - a multiscale model. J Theor Biol .

38. Schindeler, A. & Little, D. G. Recent insights into bone development, homeostasis, and repair in type 1 neurofibromatosis (NF1). *Bone* **42,** 616 (2008)

39. Atit, R. P., Crowe, M. J., Greenhalgh, D. G., Wenstrup, R. J. & Ratner, N. The Nf1 Tumor Suppressor Regulates Mouse Skin Wound Healing, Fibroblast Proliferation, and Collagen Deposited by Fibroblasts. *Journal of Investigative Dermatology* **112,** 835–842 (1999).

40. El-Hoss, J. *et al.* A Murine Model of Neurofibromatosis Type 1 Tibial Pseudarthrosis Featuring Proliferative Fibrous Tissue and Osteoclast-like Cells. *Journal of Bone and Mineral Research* **27,** 68–78 (2012).

41. Kolanczyk, M. *et al.* Modelling neurofibromatosis type 1 tibial dysplasia and its treatment with lovastatin. *BMC Medicine* **6,** (2008).

42. Geiger, M., Li, R. H. & Friess, W. Collagen sponges for bone regeneration with rhBMP-2. *Advanced Drug Delivery Reviews* **55,** 1613–1629 (2003).

43. Takahashi, Y., Yamamoto, M. & Tabata, Y. Enhanced osteoinduction by controlled release of bone morphogenetic protein-2 from biodegradable sponge composed of gelatin and b-tricalcium phosphate. *Biomaterials* **26,** 4856–4865 (2005).
